# Effects of Rumen-Protected Lysine and Methionine Supplementation on Lactation Performance in Holstein Dairy Cows: A Meta-Analysis

**DOI:** 10.3390/ani16121886

**Published:** 2026-06-18

**Authors:** Wenshuo Gao, Liyan Jiang, Yongling Bao, Xiangying Lu, Yue Hou, Lingling Li, Jiapeng Wang, Shengtao Gao

**Affiliations:** 1 College of Life Science and Technology, Inner Mongolia Normal University, Hohhot 010018, China; wenshuogao0711@163.com (W.G.); xinruli1214@163.com (L.J.); baoyongling@163.com (Y.B.); xiangyinglu2026@163.com (X.L.); houyue0089@163.com (Y.H.); li0806lingling@163.com (L.L.); 2 Key Laboratory of Biodiversity Conservation and Sustainable Utilization in Mongolian Plateau for College and University of Inner Mongolia Autonomous Region, Hohhot 010018, China

**Keywords:** rumen protected lysine, rumen protected methionine, dairy cows, meta-analysis, rumen-protected methionine analogues

## Abstract

Lysine and methionine are commonly regarded as the first limiting amino acids for lactating dairy cows, yet production responses to rumen-protected lysine (RPLys), rumen-protected methionine (RPMet), and methionine analogues vary across studies due to differences in basal diets, lactation stage, product formulation, and amino acid bioavailability. This meta-analysis synthesized evidence from 42 studies conducted in Holstein dairy cows to evaluate the effects of RPLys, RPMet, their combined supplementation, and methionine analogues on milk yield, milk fat, milk protein, milk lactose, and feed conversion-related traits. Random-effects meta-analysis showed that RPLys primarily increased milk yield, RPMet increased milk yield and milk fat percentage, combined supplementation improved milk yield, and methionine analogues increased milk yield and selected milk components. Exploratory analyses of supplementation level and Lys:Met ratio were considered hypothesis-generating only due to study-level aggregation and variation in product bioavailability. Overall, these findings support precision amino acid supplementation rather than universal strategies, and highlight the need for standardized reporting of basal diet amino acid supply and product-specific bioavailability to optimize lactation performance.

## Simple Summary

Precision amino acid nutrition is an important strategy for improving milk production efficiency while reducing unnecessary crude protein supplementation in dairy cows. Lysine and methionine are commonly considered key limiting amino acids for lactating cows, but responses to rumen-protected lysine, rumen-protected methionine, and methionine analogues vary among studies. This meta-analysis summarized evidence from 42 studies in Holstein dairy cows. Rumen-protected lysine mainly improved milk yield, whereas rumen-protected methionine increased milk yield and milk fat percentage. Combined supplementation with rumen-protected lysine and methionine improved milk yield, and methionine analogues improved milk yield and selected milk components. However, these responses depended on basal diet composition, supplementation strategy, and product characteristics. The findings support targeted amino acid balancing rather than universal supplementation and highlight the need for standardized evaluation of product bioavailability in future dairy nutrition research.

## 1. Introduction

Improving milk yield and milk composition while improving nutrient-use efficiency is a major objective of modern dairy production [1]. Protein nutrition plays a central role in determining lactation performance, but excessive crude protein supplementation is no longer considered an efficient or sustainable strategy because it increases feed costs and nitrogen excretion. Precision amino acid nutrition has therefore become an important approach for improving the efficiency of metabolizable protein use in lactating dairy cows [2]. Because free amino acids are extensively degraded by rumen microorganisms, direct supplementation of unprotected lysine and methionine is generally inefficient [3]. Rumen-protected amino acid technologies, including physical coating and chemical modification, have been developed to increase ruminal escape and post-ruminal amino acid supply [2].

Among essential amino acids, lysine and methionine are generally recognized as the two primary limiting amino acids for lactating dairy cows, although their limiting order depends on the basal diet composition and feeding system. Rumen-protected lysine (RPLys) and rumen-protected methionine (RPMet) are therefore the most extensively studied rumen-protected amino acid products in dairy nutrition [4]. Numerous studies have evaluated their effects on milk yield, milk fat percentage, milk protein percentage and milk lactose percentage in lactating dairy cows. However, the responses reported in individual studies are not always consistent [5,6]. These inconsistencies may reflect differences in basal diet composition, metabolizable protein supply, lactation stage, product type, protection technology, intestinal release, and amino acid balance. Methionine analogues, such as 2-hydroxy-4-(methylthio) butanoic acid and its isopropyl ester form, have also been evaluated as potential alternatives to RPMet [7,8]. But their relative effects on milk production and milk composition remain uncertain.

Under typical maize- and soybean meal-based feeding systems, lysine is often considered the first limiting amino acid and methionine the second limiting amino acid [9]. However, under other dietary conditions, such as pasture-based systems or diets containing high proportions of leguminous forages, this limiting order may differ [10]. The National Research Council recommended that the ratio of lysine to methionine in metabolizable protein should be approximately 3:1 to support milk protein synthesis in lactating dairy cows. However, improvements in genetic potential, changes in feeding systems, and the increasing use of rumen-protected amino acid products have created a need to re-evaluate supplementation strategies under contemporary production conditions [11].

Previous meta-analyses have assessed the effects of rumen-protected amino acids in ruminants [12,13,14]. However, existing studies have often focused on individual amino acids, specific production stages, or limited product categories. Fewer analyses have simultaneously evaluated RPLys, RPMet, combined RPLys and RPMet supplementation, and methionine analogues within the same framework. In addition, the interpretation of product comparisons remains challenging because rumen protection efficiency and bioavailability may differ substantially among commercial products and are not always assessed using standardized methods. Therefore, the present meta-analysis aimed to evaluate the effects of RPLys, RPMet, combined RPLys and RPMet supplementation, and methionine analogues on lactation performance in Holstein dairy cows. The primary outcomes were milk yield, milk fat percentage, milk protein percentage, milk lactose percentage, and feed conversion-related traits. Exploratory analyses of supplementation level and Lys:Met ratio were retained only as supplementary, hypothesis-generating analyses because of the study-level nature of the available data and the heterogeneity of product characteristics.

## 2. Materials and Methods

### 2.1. Literature Search and Eligibility Criteria

This meta-analysis was conducted and reported with reference to the Preferred Reporting Items for Systematic Reviews and Meta-Analyses (PRISMA) guidelines [15] and previous meta-analyses evaluating rumen-protected amino acids in dairy cows [13]. A systematic literature search was performed in PubMed, Web of Science and ScienceDirect to identify relevant peer-reviewed articles published between January 2000 and January 2025. The search strategy combined terms related to rumen-protected lysine, rumen-protected methionine, methionine analogues, dairy cows and lactation performance. The detailed search terms used for each database are presented in Supplementary Table S1. In addition, the reference lists of eligible articles and relevant reviews were manually screened to identify further studies that may not have been retrieved through the database search.

Studies were eligible for inclusion in the primary quantitative synthesis when they met the following criteria: (1) the experimental animals were lactating Holstein dairy cows; (2) the study included a control group and at least one treatment group receiving RPLys, RPMet, combined RPLys and RPMet, or a methionine analogue; (3) the supplemented product was described as rumen-protected, rumen-available, rumen-escape, or otherwise intended to increase post-ruminal amino acid supply; (4) at least one lactation performance outcome was reported, including milk yield, milk fat percentage, milk protein percentage, or milk lactose percentage; (5) sufficient quantitative data were available to calculate an effect size, including group means, sample size, and a measure of within-group variability or information from which variability could be derived. Feed conversion-related traits were extracted when available but were not required for inclusion.

Studies were excluded if the animals were not Holstein dairy cows, if no appropriate control group was included, if the supplemented amino acids were not protected or not intended to escape ruminal degradation, or if the effect of the amino acid product could not be separated from the effects of other dietary or management interventions. Studies were also excluded when relevant outcome data were not reported, could not be extracted from the text, tables or figures, or could not be obtained or calculated from the available information. When multiple reports appeared to describe the same experimental dataset, only the most complete report was retained to avoid duplicate inclusion.

### 2.2. Data Extraction

Data were extracted independently from each eligible study using a standardized data extraction form. The extracted information included the type of amino acid product, supplementation level, experimental design, number of animals, treatment groups, and reported outcomes. The outcomes extracted for meta-analysis included milk yield, milk fat percentage, milk protein percentage, milk lactose percentage and feed conversion-related traits when available.

For each outcome, treatment and control group means, sample sizes and measures of variability were extracted. When standard deviations were not directly reported, they were calculated from reported standard errors or standard errors of the mean according to standard statistical procedures [16]. If only the standard error was reported, the standard deviation was calculated by multiplying the standard error by the square root of the corresponding sample size. When studies reported multiple treatment levels of the same amino acid product, each treatment level was treated as a separate comparison with the corresponding control group. For multi-arm studies sharing a single control group, the control group sample size was adjusted where appropriate to reduce the risk of double-counting in pooled analyses.

Supplementation levels were extracted as reported in the original studies and converted to a common percentage basis when possible. However, reported supplementation levels were not uniformly adjusted for true absorbed amino acid supply because independent bioavailability estimates obtained using a common methodology were not available for all products. Therefore, treatment effects should be interpreted as responses to reported supplementation strategies rather than precise responses to absorbed lysine or methionine supply.

### 2.3. Quality Assessment

The methodological quality and reporting completeness of the included studies were assessed independently by two reviewers, and disagreements were resolved by discussion. The assessment focused on factors likely to affect the reliability of extracted effect sizes: reporting of within-group variability, absence of duplicate reporting, and sample size. Studies received 5 points if within-group SD or SE values were reported or could be clearly derived, and 0 points otherwise. Studies received 5 points when no duplicate reporting of the same dataset was detected. Sample size was scored as follows: >150 animals, 5 points; 100 to 149 animals, 4 points; 70 to 99 animals, 3 points; 30 to 69 animals, 2 points; and <30 animals, 1 point. Total scores were classified as low- (≤6), moderate- (7–11), or high-quality (≥12). Detailed quality scores are presented in Supplementary Table S2.

### 2.4. Statistical Analysis

The effects of RPLys, RPMet, combined RPLys and RPMet, and methionine analogues on lactation performance were evaluated by meta-analysis. Because the included studies differed in basal diet composition, lactation stage, product formulation, supplementation level, and experimental design, pooled estimates were calculated using a random-effects model. Effect sizes were expressed as standardized mean differences (SMDs) with 95% confidence intervals (CIs). A positive SMD indicated that the treatment mean was higher than the control mean, whereas a negative SMD indicated a lower treatment mean.

The SMD was selected because the included studies differed in design, product formulation, supplementation level, and variability structure. Statistical significance was determined using the 95% CI of the pooled estimate and the associated *p*-value. Effects were considered statistically significant when the 95% CI did not include zero. The magnitude of the effect was interpreted using conventional standardized effect-size thresholds, with absolute SMDs of approximately 0.2, 0.5, and 0.8 considered small, moderate, and large effects, respectively. These thresholds were used only as general interpretive guides and not as substitutes for biological or production relevance.

Between-study heterogeneity was assessed using Cochran’s Q-test and the I^2^ statistic. I^2^ values greater than 50% were considered to indicate substantial heterogeneity. Subgroup analyses were conducted according to amino acid supplementation category: RPLys alone, RPMet alone, combined RPLys and RPMet, and methionine analogues. When at least 10 comparisons were available for an outcome, sensitivity analysis was performed by sequentially removing individual studies to evaluate their influence on the pooled estimate and heterogeneity. Potential publication bias was assessed by visual inspection of funnel plots when sufficient comparisons were available.

An important limitation of the analysis is that rumen-protected amino acid products differ substantially in coating technology, ruminal escape, intestinal release, and true bioavailability. Manufacturer-reported bioavailability values are not always generated using standardized methodologies and may therefore not be directly comparable across products. Because independent bioavailability estimates obtained using a common methodology were unavailable for all products, the present analysis did not adjust all treatments to a common bioavailable amino acid supply. This issue is particularly relevant for methionine analogue products and may have contributed to between-study heterogeneity. Only seven of the included English studies reported measured bioavailability data, whereas the remaining trials lacked empirical measurements for this parameter. Accordingly, bioavailability was not adopted as a criterion for literature screening and methodological quality assessment in the present meta-analysis.

Exploratory analyses of supplementation level and Lys:Met ratio were conducted using aggregated study-level or treatment-level data and are presented only in the Supplementary Materials. These analyses were considered descriptive and hypothesis-generating because they were not performed using cow-level data and were affected by differences in basal diet amino acid supply, product formulation, protection technology, and bioavailability assumptions. Therefore, they were not used to support the main conclusions of the study. Meta-analyses were conducted using Review Manager version 5.4.

## 3. Results

### 3.1. Study Selection and Characteristics

**Figure 1:**
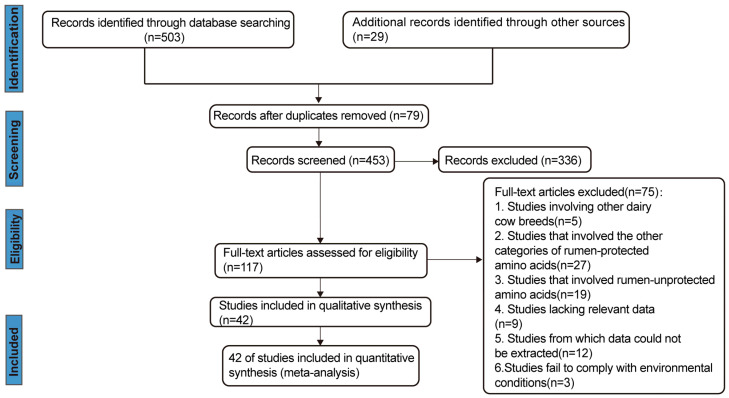
Flow diagram illustrating the process of study identification, screening, eligibility assessment, and inclusion in the meta-analysis. Forty-two studies conducted in Holstein dairy cows were included in the primary quantitative synthesis. Three eligible studies were identified during screening but were excluded from the primary meta-analysis because the number of studies was insufficient for reliable subgroup analysis.

The database search identified 503 potentially relevant records from PubMed, Web of Science, and ScienceDirect. An additional 29 records were identified through manual screening of reference lists and relevant publications. After duplicate removal, 453 records remained for title and abstract screening. Among these records, 336 were excluded because they were unrelated to the objectives of this meta-analysis, did not evaluate rumen-protected amino acids, dairy breeds non-Holstein dairy cows, or did not report relevant lactation performance outcomes. The remaining 117 full-text articles were assessed for eligibility. After full-text review, 42 studies met the inclusion criteria and were included in the primary quantitative synthesis (Figure 1).

All studies included in the primary meta-analysis were conducted in Holstein dairy cows thermoneutral conditions. Studies conducted specifically were not included in the primary quantitative synthesis because only three eligible reports were identified, which was insufficient to support a reliable subgroup analysis. The main characteristics of the included studies, including type of rumen-protected amino acids, supplementation level, sample size, treatment structure, and reported outcomes are shown in Table 1.

Among the 42 included studies, the supplemented products included rumen-protected lysine (RPLys), rumen-protected methionine (RPMet), combined RPLys and RPMet, and methionine analogues or analogue-based products. Fourteen studies evaluated methionine analogues or methionine analogue-based products. According to the quality assessment, six studies were classified as high-quality, 30 as moderate-quality, and six as low-quality. The main factors contributing to lower quality scores were small sample size and incomplete reporting of within-group variability (Supplementary Table S2).

Sensitivity analyses indicated that individual studies contributed to heterogeneity for some outcomes, particularly milk yield and milk fat percentage. For milk yield, exclusion of one influential study [37] reduced the I^2^ value from 90% to 63%, indicating that this study contributed substantially to between-study heterogeneity (Supplementary Figures S1 and S2). For milk fat percentage, exclusion of another influential study [51] reduced the I^2^ value from 22% to 0% (Supplementary Figures S3 and S4).

### 3.2. Effects of RPLys Supplementation Alone on Lactation Performance

**Figure 2:**
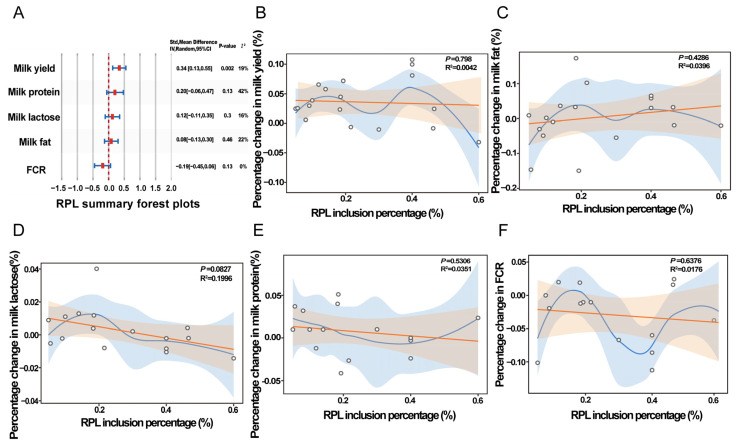
Effects of rumen-protected lysine (RPLys) supplementation alone on lactation performance and feed conversion-related traits in Holstein dairy cows. (**A**) Forest plot showing pooled standardized mean differences (SMDs) with 95% confidence intervals (CIs) for milk yield, milk protein percentage, milk lactose percentage, milk fat percentage, and feed conversion-related traits. (**B**) Exploratory association between RPLys inclusion level and percentage change in milk yield. (**C**) Exploratory association between RPLys inclusion level and percentage change in milk fat percentage. (**D**) Exploratory association between RPLys inclusion level and percentage change in milk lactose percentage. (**E**) Exploratory association between RPLys inclusion level and percentage change in milk protein percentage. (**F**) Exploratory association between RPLys inclusion level and percentage change in feed conversion-related traits. The funnel plot is presented in Figure S5, and the forest plots are shown in Figures S6–S10. In plot (**A**), horizontal blue lines refer to 95% CIs and solid red squares denote pooled SMDs. Blue fitted lines in (**B**–**F**) illustrate linear associations between amino acid supplementation levels and lactation traits. A positive SMD indicates that the treatment mean was higher than the control mean, whereas a negative SMD indicates that the treatment mean was lower than the control mean. Dose–response panels are descriptive and hypothesis-generating only. The dose–response regression fitting only confirmed a significant correlation between rumen-protected amino acid supplementation level and production response, but did not verify the accuracy of the optimal dosage. Substantial heterogeneity was observed in the interpretation of dose–response relationships and experimental implementation across studies, which may be attributed to variations in the bioavailability of commercial rumen-protected products and differences in basal diet formulation.

The pooled effects of RPLys supplementation alone were summarized using a random-effects forest plot (Figure 2A). RPLys supplementation significantly increased milk yield compared with the control treatment (SMD = 0.34; 95% CI: 0.13 to 0.55; *p* = 0.002; I^2^ = 19%). In contrast, no significant pooled effects were observed for milk protein percentage (SMD = 0.20; 95% CI: −0.06 to 0.47; *p* = 0.13; I^2^ = 42%), milk lactose percentage (SMD = 0.12; 95% CI: −0.11 to 0.35; *p* = 0.30; I^2^ = 16%), milk fat percentage (SMD = 0.08; 95% CI: −0.13 to 0.30; *p* = 0.46; I^2^ = 22%), or feed conversion-related traits (SMD = −0.19; 95% CI: −0.45 to 0.06; *p* = 0.13; I^2^ = 0%). These results suggest that, under the conditions represented by the included studies, RPLys supplementation was mainly associated with improved milk yield, whereas its effects on milk composition and feed conversion-related traits were not statistically significant.

Exploratory dose–response analyses based on aggregated treatment-level data were conducted for milk yield responses (Figure 2B), milk fat percentage responses (Figure 2C), milk lactose percentage responses (Figure 2D), milk protein percentage responses (Figure 2E), and feed conversion-related traits (Figure 2F). These analyses showed variable numerical responses across RPLys inclusion levels, but the fitted associations were weak and should not be interpreted as evidence of an optimal supplementation level. Differences among studies in basal diet composition, lactation stage, product formulation, protection technology, and estimated amino acid bioavailability may have contributed to the observed variation.

### 3.3. The Effects of Adding RPMet Alone on Dairy Cow Production Performance

**Figure 3:**
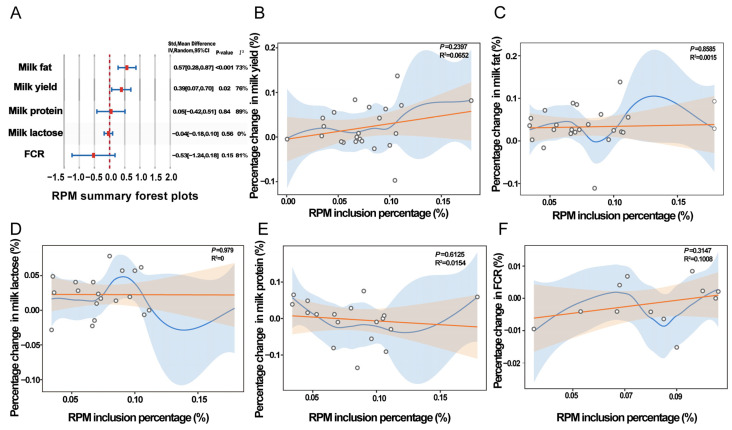
Effects of rumen-protected methionine (RPMet) supplementation alone on lactation performance and feed conversion-related traits in Holstein dairy cows. (**A**) Forest plot showing pooled standardized mean differences (SMDs) with 95% confidence intervals (CIs) for milk fat percentage, milk yield, milk protein percentage, milk lactose percentage, and feed conversion-related traits. (**B**) Exploratory association between RPMet inclusion level and percentage change in milk yield. (**C**) Exploratory association between RPMet inclusion level and percentage change in milk fat percentage. (**D**) Exploratory association between RPMet inclusion level and percentage change in milk lactose percentage. (**E**) Exploratory association between RPMet inclusion level and percentage change in milk protein percentage. (**F**) Exploratory association between RPMet inclusion level and percentage change in feed conversion-related traits. The funnel plot is presented in Figure S5, and the forest plots are shown in Figures S6–S10. In plot (**A**), horizontal blue lines refer to 95% CIs and solid red squares denote pooled SMDs. Blue fitted lines in (**B**–**F**) illustrate linear associations between amino acid supplementation levels and lactation traits. A positive SMD indicates that the treatment mean was higher than the control mean, whereas a negative SMD indicates that the treatment mean was lower than the control mean. Dose–response panels are descriptive and hypothesis-generating only. The dose–response regression fitting only confirmed a significant correlation between rumen-protected amino acid supplementation level and production response, but did not verify the accuracy of the optimal dosage. Substantial heterogeneity was observed in the interpretation of dose–response relationships and experimental implementation across studies, which may be attributed to variations in the bioavailability of commercial rumen-protected products and differences in basal diet formulation.

The pooled effects of RPMet supplementation alone were evaluated using a random-effects forest plot (Figure 3A). RPMet supplementation significantly increased milk yield compared with the control treatment (SMD = 0.39; 95% CI: 0.07 to 0.70; *p* = 0.02; I^2^ = 76%). RPMet supplementation also significantly increased milk fat percentage (SMD = 0.57; 95% CI: 0.28 to 0.87; *p* < 0.001; I^2^ = 73%). These findings indicate that RPMet supplementation was associated with improvements in both milk production and milk fat concentration. No significant pooled effects of RPMet supplementation were detected for milk protein percentage (SMD = 0.05; 95% CI: −0.42 to 0.51; *p* = 0.84; I^2^ = 89%), milk lactose percentage (SMD = −0.04; 95% CI: −0.18 to 0.10; *p* = 0.56; I^2^ = 0%), or feed conversion-related traits (SMD = −0.53; 95% CI: −1.24 to 0.18; *p* = 0.15; I^2^ = 81%). Therefore, the overall evidence from the included studies supports a positive effect of RPMet on milk yield and milk fat percentage, whereas its effects on other lactation performance variables were not statistically significant.

Exploratory analyses evaluating associations between RPMet inclusion level and percentage changes in lactation traits were performed for milk yield responses (Figure 3B), milk fat percentage responses (Figure 3C), milk lactose percentage responses (Figure 3D), milk protein percentage responses (Figure 3E), and feed conversion-related traits (Figure 3F). Because these analyses were based on aggregated study-level or treatment-level data, and because the included products differed in coating technology, ruminal escape, intestinal release, and estimated bioavailability, these results should be regarded as descriptive and hypothesis-generating only.

### 3.4. Effects of Combined RPLys and RPMet Supplementation on Lactation Performance

**Figure 4:**
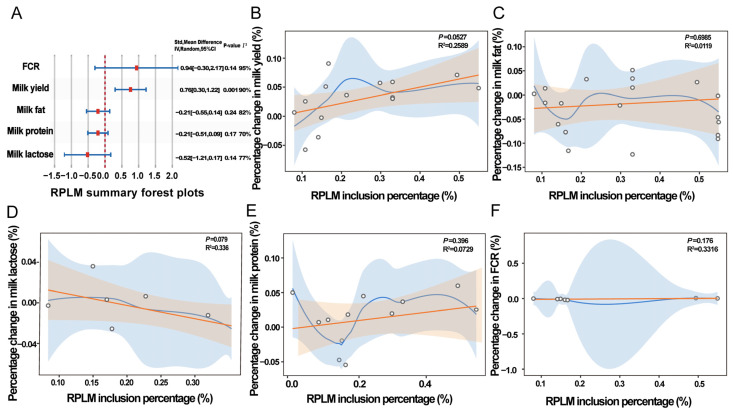
Effects of combined supplementation with rumen-protected lysine (RPLys) and rumen-protected methionine (RPMet) on lactation performance and feed conversion-related traits in Holstein dairy cows. (**A**) Forest plot showing pooled standardized mean differences (SMDs) with 95% confidence intervals (CIs) for feed conversion-related traits, milk yield, milk fat percentage, milk protein percentage, and milk lactose percentage. (**B**) Exploratory association between combined RPLys and RPMet inclusion level and percentage change in milk yield. (**C**) Exploratory association between combined RPLys and RPMet inclusion level and percentage change in milk fat percentage. (**D**) Exploratory association between combined RPLys and RPMet inclusion level and percentage change in milk lactose percentage. (**E**) Exploratory association between combined RPLys and RPMet inclusion level and percentage change in milk protein percentage. (**F**) Exploratory association between combined RPLys and RPMet inclusion level and percentage change in feed conversion-related traits. The funnel plot is presented in Figure S5, and the forest plots are shown in Figures S6–S10. In plot (**A**), horizontal blue lines refer to 95% CIs and solid red squares denote pooled SMDs. Blue fitted lines in (**B**–**F**) illustrate linear associations between amino acid supplementation levels and lactation traits. A positive SMD indicates that the treatment mean was higher than the control mean, whereas a negative SMD indicates that the treatment mean was lower than the control mean. Dose–response panels are descriptive and hypothesis-generating only. The dose–response regression fitting only confirmed a significant correlation between rumen-protected amino acid supplementation level and production response, but did not verify the accuracy of the optimal dosage. Substantial heterogeneity was observed in the interpretation of dose–response relationships and experimental implementation across studies, which may be attributed to variations in the bioavailability of commercial rumen-protected products and differences in basal diet formulation.

The pooled effects of combined supplementation with RPLys and RPMet were summarized using a random-effects forest plot (Figure 4A). Combined supplementation significantly increased milk yield compared with the control treatment (SMD = 0.76; 95% CI: 0.30 to 1.22; *p* = 0.001; I^2^ = 90%). This effect size was numerically larger than that observed for RPLys or RPMet supplementation alone, suggesting that simultaneous supplementation may be beneficial when both amino acids are limiting or when their balance in metabolizable protein is improved. However, the high heterogeneity indicates that the magnitude of response differed substantially among studies. No significant pooled effects of combined RPLys and RPMet supplementation were observed for feed conversion-related traits (SMD = 0.94; 95% CI: −0.30 to 2.17; *p* = 0.14; I^2^ = 95%), milk fat percentage (SMD = −0.21; 95% CI: −0.55 to 0.14; *p* = 0.24; I^2^ = 82%), milk protein percentage (SMD = −0.21; 95% CI: −0.51 to 0.09; *p* = 0.17; I^2^ = 70%), or milk lactose percentage (SMD = −0.52; 95% CI: −1.21 to 0.17; *p* = 0.14; I^2^ = 77%). Thus, the main response to combined supplementation in the present analysis was an increase in milk yield, whereas effects on milk composition and feed conversion-related traits were not consistently detected across studies.

Exploratory analyses of combined RPLys and RPMet inclusion level were conducted for milk yield responses (Figure 4B), milk fat percentage responses (Figure 4C), milk lactose percentage responses (Figure 4D), milk protein percentage responses (Figure 4E), and feed conversion-related traits (Figure 4F). These analyses showed variable numerical responses across inclusion levels, but the associations were based on a limited number of aggregated comparisons and were not sufficiently robust to define a recommended dose. Therefore, these exploratory panels should be interpreted cautiously and considered only as preliminary evidence for future dose–response studies.

### 3.5. Exploratory Association Between Supplemental Lys:Met Ratio and Lactation Performance

**Figure 5:**
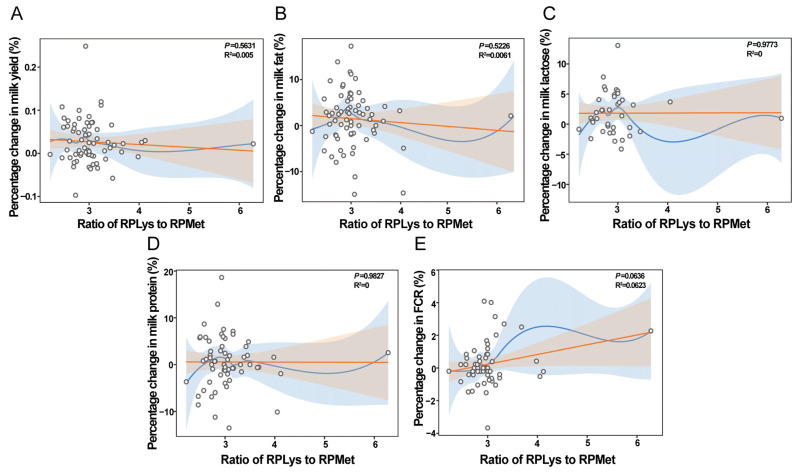
Exploratory associations between supplemental Lys:Met ratio and lactation performance or feed conversion-related traits in Holstein dairy cows. (**A**) Association between supplemental Lys:Met ratio and percentage change in milk yield. (**B**) Association between supplemental Lys:Met ratio and percentage change in milk fat percentage. (**C**) Association between supplemental Lys:Met ratio and percentage change in milk lactose percentage. (**D**) Association between supplemental Lys:Met ratio and percentage change in milk protein percentage. (**E**) Association between supplemental Lys:Met ratio and percentage change in feed conversion-related traits. The funnel plot is presented in Figure S5, and the forest plots are shown in Figures S6–S10. Blue fitted lines in (**A**–**E**) illustrate linear associations between amino acid supplementation levels and lactation traits. The ratio was calculated from supplemental amino acid inputs rather than the measured or model-predicted metabolizable lysine and methionine supply. Therefore, these analyses are descriptive and hypothesis-generating only and should not be used to define an optimal Lys:Met ratio. The dose–response regression fitting only confirmed a significant correlation between rumen-protected amino acid supplementation level and production response, but did not verify the accuracy of the optimal dosage. Substantial heterogeneity was observed in the interpretation of dose–response relationships and experimental implementation across studies, which may be attributed to variations in the bioavailability of commercial rumen-protected products and differences in basal diet formulation.

The association between supplemental Lys:Met ratio and lactation performance was evaluated as an exploratory analysis. The exploratory panels included percentage changes in milk yield (Figure 5A), milk fat percentage (Figure 5B), milk lactose percentage (Figure 5C), milk protein percentage (Figure 5D), and feed conversion-related traits (Figure 5E). These analyses suggested that numerical responses varied across supplemental Lys:Met ratios, but the observed patterns should not be interpreted as direct evidence of an optimal Lys:Met ratio.

Several factors limit the interpretation of these exploratory associations. First, the ratio was calculated from supplemental amino acid inputs rather than from measured or model-predicted metabolizable lysine and methionine supply. Second, basal diet amino acid composition differed among studies and was not consistently reported in sufficient detail to estimate total absorbable amino acid supply. Third, product-specific ruminal protection, intestinal release, and true bioavailability were not assessed using a standardized methodology across all included products. Therefore, the Lys:Met ratio analysis was retained only as a descriptive, hypothesis-generating component of the study and was not used to make definitive recommendations.

### 3.6. Effects of Methionine Analogue Supplementation on Lactation Performance

**Figure 6:**
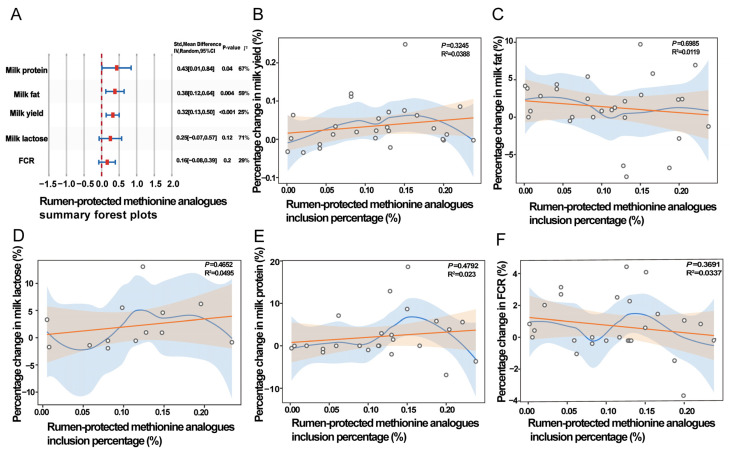
Effects of methionine analogue or analogue-based product supplementation on lactation performance and feed conversion-related traits in Holstein dairy cows. (**A**) Forest plot showing pooled standardized mean differences (SMDs) with 95% confidence intervals (CIs) for milk protein percentage, milk fat percentage, milk yield, milk lactose percentage, and feed conversion-related traits. (**B**) Exploratory association between methionine analogue inclusion level and percentage change in milk yield. (**C**) Exploratory association between methionine analogue inclusion level and percentage change in milk fat percentage. (**D**) Exploratory association between methionine analogue inclusion level and percentage change in milk lactose percentage. (**E**) Exploratory association between methionine analogue inclusion level and percentage change in milk protein percentage. (**F**) Exploratory association between methionine analogue inclusion level and percentage change in feed conversion-related traits. The funnel plot is presented in Figure S5, and the forest plots are shown in Figures S6–S10. In plot (**A**), horizontal blue lines refer to 95% CIs and solid red squares denote pooled SMDs. Blue fitted lines in (**B**–**F**) illustrate linear associations between amino acid supplementation levels and lactation traits. A positive SMD indicates that the treatment mean was higher than the control mean, whereas a negative SMD indicates that the treatment mean was lower than the control mean. Results should be interpreted as responses to reported supplementation strategies rather than as precise responses to absorbed methionine supply because product-specific bioavailability was not standardized across studies. The dose–response regression fitting only confirmed a significant correlation between rumen-protected amino acid supplementation level and production response, but did not verify the accuracy of the optimal dosage. Substantial heterogeneity was observed in the interpretation of dose–response relationships and experimental implementation across studies, which may be attributed to variations in the bioavailability of commercial rumen-protected products and differences in basal diet formulation.

The pooled effects of methionine analogue supplementation were summarized using a random-effects forest plot (Figure 6A). Methionine analogues significantly increased milk yield compared with the control treatment (SMD = 0.32; 95% CI: 0.13 to 0.50; *p* < 0.001; I^2^ = 25%). Methionine analogue supplementation also significantly increased milk fat percentage (SMD = 0.38; 95% CI: 0.12 to 0.64; *p* = 0.004; I^2^ = 59%) and milk protein percentage (SMD = 0.43; 95% CI: 0.01 to 0.84; *p* = 0.04; I^2^ = 67%). No significant pooled effects were observed for milk lactose percentage (SMD = 0.25; 95% CI: −0.07 to 0.57; *p* = 0.12; I^2^ = 71%) or feed conversion-related traits (SMD = 0.16; 95% CI: −0.08 to 0.39; *p* = 0.20; I^2^ = 29%).

These results indicate that methionine analogues may improve selected lactation performance traits, including milk yield, milk fat percentage, and milk protein percentage. However, comparisons between RPMet and methionine analogues should be interpreted cautiously because most available evidence was derived from comparisons across different studies rather than direct head-to-head comparisons within the same experimental design.

Exploratory dose–response analyses for methionine analogues were conducted for milk yield responses (Figure 6B), milk fat percentage responses (Figure 6C), milk lactose percentage responses (Figure 6D), milk protein percentage responses (Figure 6E), and feed conversion-related traits (Figure 6F). These analyses showed variable numerical responses across inclusion levels, but their interpretation is limited by differences among analogue products, including chemical form, ruminal metabolism, intestinal availability, and manufacturer-reported bioavailability. Because independent estimates of bioavailability obtained using a common methodology were not available for all products, these exploratory panels should be interpreted as descriptive responses to reported supplementation strategies rather than as precise responses to absorbed methionine supply.

## 4. Discussion

The present meta-analysis synthesized evidence from 42 studies conducted in lactating Holstein dairy cows and showed that the productive responses to rumen-protected amino acid supplementation were dependent on amino acid source, supplementation strategy, and the nutritional context of the basal diet. Overall, RPLys supplementation was associated primarily with increased milk yield, RPMet supplementation increased both milk yield and milk fat percentage, combined RPLys and RPMet supplementation improved milk yield, and methionine analogues improved milk yield and selected milk components. However, these responses were not uniform across outcomes, and the magnitude of response varied among studies. This variation indicates that the biological effects of supplemental Lys and Met cannot be interpreted solely as a function of the amount of product added to the diet, but should instead be considered in relation to basal diet amino acid supply, lactation stage, production level, amino acid balance, and product-specific ruminal protection and post-ruminal availability.

RPLys supplementation significantly increased milk yield, which is consistent with the concept that Lys is frequently one of the first limiting amino acids in dairy cow diets, particularly in maize- and soybean meal-based feeding systems [13,58]. A previous meta-analysis also reported positive production responses to RPLys supplementation, especially when the basal diet was more likely to be limited in absorbable Lys [13]. From a biological perspective, supplemental RPLys may improve the profile of absorbed essential amino acids and thereby support greater use of metabolizable protein for milk synthesis [59]. Nevertheless, the present analysis did not detect consistent pooled effects of RPLys on milk fat percentage, milk protein percentage, milk lactose percentage, or feed conversion-related traits. This suggests that the primary detectable response to RPLys in the available dataset was increased milk output rather than a broad improvement across milk composition variables. The absence of consistent effects on milk components may reflect differences among studies in basal amino acid adequacy, energy supply, rumen-undegradable protein sources, and stage of lactation. It may also indicate that Lys supplementation alone is most effective when Lys is clearly limiting, whereas responses may be attenuated when other amino acids, energy supply, or overall metabolizable protein supply become co-limiting.

Methionine has multiple biological functions that may explain its positive effects on lactation performance. It plays a central role in one-carbon metabolism and cellular methylation reactions [60], and is also involved in antioxidant defence, immune regulation and mammary epithelial cell function. The mTOR signalling pathway is an important intracellular nutrient-sensing mechanism regulating protein synthesis and cellular metabolism [61], and Met can promote molecular processes associated with milk synthesis in mammary epithelial cells [62]. Met may also influence milk fat synthesis through mechanisms related to methyl donor availability, lipid metabolism, and the regulation of mammary lipogenic pathways [63]. In agreement with these biological roles, the present meta-analysis showed that RPMet significantly increased milk yield and milk fat percentage. This result is consistent with recent quantitative evidence indicating that RPMet supplementation can improve milk fat responses in transition and lactating dairy cows [64]. However, RPMet did not significantly improve milk protein percentage, milk lactose percentage, or feed conversion-related traits in the present analysis. Therefore, the response to RPMet should be interpreted as trait-specific rather than universal. The comparatively consistent response in milk fat percentage may indicate that Met supply has a stronger detectable influence on lipid-related lactation responses than on some other milk composition traits under the conditions represented in the included studies.

Combined supplementation with RPLys and RPMet significantly increased milk yield, supporting the nutritional principle that balancing the supply of the two major limiting amino acids is important for optimizing metabolizable protein utilization in lactating dairy cows. The National Research Council emphasized the importance of an appropriate balance between Lys and Met in metabolizable protein to support milk protein synthesis [10]. In practical ration formulation, simultaneous supplementation may be beneficial when both amino acids are limiting or when supplementation improves the balance of absorbed amino acids reaching the small intestine. However, the response to combined RPLys and RPMet supplementation was not consistently superior across all lactation outcomes, and no significant pooled effects were observed for milk fat percentage, milk protein percentage, milk lactose percentage, or feed conversion-related traits. This finding indicates that the benefits of combined supplementation depend strongly on the limiting amino acid status of the basal diet. Irawan et al. [13] also reported that supplementing RPLys and RPMet to amino acid-adequate diets did not necessarily further improve productive performance. Therefore, the present results support precision amino acid balancing rather than routine supplementation without prior evaluation of dietary amino acid supply. In dairy practice, the predicted supply of metabolizable Lys and Met should be assessed before supplementation, particularly in relation to dietary crude protein level, rumen-undegradable protein source, forage base, and expected milk yield.

Methionine analogues, including 2-hydroxy-4-(methylthio)butanoic acid and its isopropyl ester form, were included in this meta-analysis because they are used as Met-related supplements in dairy cow nutrition and may differ from physically protected RPMet in ruminal metabolism, absorption route, and metabolic conversion. The present results showed that methionine analogue supplementation significantly increased milk yield, milk fat percentage, and milk protein percentage. These responses may be partly related to the ability of Met-related compounds to influence mammary protein synthesis pathways; for example, HMBi has been reported to affect β-casein expression and molecular pathways associated with milk protein synthesis in bovine mammary epithelial cells [65]. However, methionine analogues should not be interpreted as directly equivalent to physically protected RPMet products. Their chemical form, ruminal degradation or metabolism, intestinal availability, and conversion efficiency may differ substantially among products. These product-specific differences are important because most comparisons in the present meta-analysis were made across studies rather than through direct head-to-head comparisons within the same experimental design [64]. Therefore, the observed responses should be interpreted as effects of reported supplementation strategies rather than evidence that methionine analogues are biologically equivalent or superior to RPMet. Direct comparative studies under standardized dietary conditions are needed before strong conclusions can be made regarding the relative efficacy of different Met sources.

The interpretation of methionine analogue responses is also closely linked to a broader limitation of rumen-protected amino acid research: the uncertainty surrounding bioavailability estimates. Rumen-protected products differ markedly in coating technology, ruminal escape, intestinal release, intestinal digestibility, and true post-ruminal amino acid availability. In many published studies, bioavailability values are derived from manufacturer reports or from product-specific methods that are not standardized across commercial sources. This creates a potential source of bias when products are compared using reported supplementation levels or assumed bioavailable amino acid supply. If true bioavailability is lower than assumed, the biological response may be underestimated relative to the intended absorbed dose. Conversely, if treatments are interpreted as delivering equivalent amounts of absorbable Lys or Met when they do not, apparent differences among products may reflect inaccurate assumptions about the delivered amino acid rather than true differences in biological efficacy. This limitation is particularly important for methionine analogues and coated Met products, because their ruminal and post-ruminal behaviour can differ substantially. Consequently, the present meta-analysis did not attempt to standardize all treatments to a common absorbed amino acid supply. Accordingly, future studies and meta-analyses should prioritize independent and standardized bioavailability assessments under comparable experimental conditions, so that product comparisons are based on verified absorbable Lys and Met supply rather than manufacturer-reported estimates.

The exploratory analyses of supplementation level and supplemental Lys:Met ratio were retained only as descriptive, hypothesis-generating information. Although the Lys:Met balance in metabolizable protein is an important concept in dairy nutrition, and a ratio close to 3:1 has been widely discussed as a reference target [10], the present dataset was not sufficient to define an optimal ratio. The ratio evaluated in this study was calculated from supplemental amino acid inputs rather than from measured or model-predicted metabolizable Lys and Met supply. In addition, basal diet amino acid composition, rumen-undegradable protein source, crude protein concentration, lactation stage, product formulation, and bioavailability assumptions varied among studies. Therefore, the exploratory Lys:Met analysis should not be used to revise feeding recommendations or define a fixed target ratio. Future research integrating measured or model-predicted metabolizable amino acid supply with animal-level performance data will be required to refine amino acid balance recommendations for high-producing dairy cows.

Several limitations should be acknowledged. First, substantial heterogeneity was observed for several outcomes, likely reflecting variation in basal diet composition, forage-to-concentrate ratio, crude protein level, rumen-undegradable protein source, lactation stage, parity, milk production level, and study design. Second, the effective delivery of absorbable Lys and Met could not be standardized across products because independent bioavailability estimates obtained using a common methodology were not available for all amino acid sources. Third, the exploratory analyses were based on aggregated study-level or treatment-level data rather than cow-level observations, and they were therefore not suitable for establishing optimal inclusion rates or definitive Lys:Met targets. Fourth, some studies had small sample sizes or incomplete reporting of within-group variability, which may affect the precision of effect-size estimates. Finally, feed conversion-related traits were not reported consistently across studies, and definitions of these traits varied, limiting the strength of conclusions regarding nutrient-use efficiency. Collectively, these limitations indicate that responses to rumen-protected amino acid supplementation should be interpreted in relation to dietary context, product characteristics, and the evidentiary limits of the available literature.

## 5. Conclusions

This meta-analysis demonstrates that rumen-protected amino acid supplementation can improve selected lactation traits in Holstein dairy cows, with effects dependent on amino acid source, supplementation strategy, and basal diet composition. Rumen-protected lysine primarily increased milk yield, rumen-protected methionine enhanced milk yield and milk fat percentage, combined supplementation of lysine and methionine increased milk yield, and methionine analogues improved milk yield together with selected milk components. However, these responses were not uniform across outcomes and should be interpreted cautiously due to between-study heterogeneity, variations in basal diet amino acid supply, and uncertainty regarding product-specific ruminal protection, intestinal digestibility, and bioavailability estimates. Exploratory analyses of dose and Lys:Met ratio were considered hypothesis-generating only and do not justify definitive recommendations regarding optimal inclusion rates or fixed target ratios. Collectively, the findings support the use of precision amino acid balancing rather than universal supplementation strategies. Future research should employ standardized bioavailability assessment, report basal diet amino acid supply in detail, and conduct direct comparative and animal-level dose–response experiments to develop reliable, efficient, and sustainable feeding recommendations for modern dairy production.

## Figures and Tables

**Table 1:** Characteristics of the included studies.

Study	Type of Rumen-Protected Amino Acids	Supplemental Amino Acid Percentage (%)	Sample Size	Groups	Outcomes
[17]	MHA	0.062% 0.128% 0.1875%	60	Control and MHA	Milk yield, Milk fat percentage and Milk lactose percentage, FCR
[18]	RPMet	0.096%	40	Control and RPMet	Milk yield, Milk fat percentage and Milk lactose percentage, Milk protein percentage
[19]	HMBi	0.151%	24	Control and HMBi	Milk yield, Milk fat percentage and Milk lactose percentage, Milk protein percentage
[20]	Mepron and Smartamine M	0.021% 0.042% 0.042%	16	Control, Mepron and Smartamine M	Milk yield, Milk fat percentage and Milk lactose percentage
[7]	RPMet, HMBi and HMB	0.061%, 0.165%, 0.06% + 0.1%	70	Control and HMBi, RPMet, RPM + HMB	Milk yield, Milk fat percentage and Milk lactose percentage, FCR
[21]	Smartamine M	0.199%	40	Control and Smartamine M	Milk yield, Milk fat percentage and Milk lactose percentage, Milk protein percentage
[22]	RPLys	0.40%	75	Control and RPLys	Milk yield, Milk fat percentage and Milk lactose percentage, Milk protein percentage, FCR
[23]	RPMet, RPLys	0.106% 0.463%	72	Control, RPMet and RPLys	Milk yield, Milk fat percentage and Milk lactose percentage, FCR
[24]	RPLys	0.216% 0.193%	68	Control and RPLys	Milk yield, Milk fat percentage and Milk lactose percentage, Milk protein percentage
[25]	RPMet, RPLys	0.045% 0.449%	200	Control and RPMet + RPLys	Milk yield, Milk fat percentage and Milk lactose percentage, FCR
[26]	RPMet	0.071%	166	Control and RPMet	Milk yield, Milk fat percentage and Milk lactose percentage, Milk protein percentage
[27]	RPMet, RPLys	0.422% 0.127%	48	Control, RPMet and RPLys	Milk yield, Milk fat percentage and Milk lactose percentage, FCR
[28]	RPLys, RPMet + RPLys	0.091% 0.058%	8	Control, RPLys and RPMet + RPLys	Milk yield, Milk fat percentage and Milk lactose percentage, FCR
[29]	RPMet	0.068%	96	Control and RPMet	Milk yield, Milk fat percentage and Milk lactose percentage, Milk protein percentage
[30]	NALM	0.059% 0.117% 0.238%	48	Control and NALM	Milk yield, Milk fat percentage and Milk lactose percentage, Milk protein percentage, FCR
[31]	RPLys	0.466%	32	Control and RPLys	Milk yield, Milk fat percentage and Milk lactose percentage
[32]	RPLys	0.3% 0.6%	36	Control and RPLys	Milk yield, Milk fat percentage and Milk lactose percentage, Milk protein percentage, FCR
[33]	RPMet, RPLys	0.119% 0.179%	144	Control, RPMet and RPLys	Milk yield, Milk fat percentage and Milk protein percentage
[34]	RPMet, RPLys	0.0804%	104	Control, RPMet and RPLys	Milk yield, Milk fat percentage and Milk protein percentage
[35]	HMBi	0.13%	40	Control and HMBi	Milk yield, Milk fat percentage and Milk lactose percentage, Milk protein percentage
[36]	RPLys	0.05% 0.10% 0.14%	32	Control and RPLys	Milk yield, Milk fat percentage and Milk lactose percentage, Milk protein percentage
[37]	RPMet, RPLys	0.122% + 0.091%	96	Control and RPMet + RPLys	Milk yield, Milk fat percentage and Milk protein percentage, FCR
[38]	HMBi	0.54% 0.10%	57	Control and HMBi	Milk yield, Milk fat percentage and Milk lactose percentage, Milk protein percentage, FCR
[39]	HMB	0.13% 0.2%	48	Control and HMB	Milk yield, Milk fat percentage and Milk lactose percentage
[40]	NALM	0.132%	18	Control and NALM	Milk yield, Milk fat percentage and Milk lactose percentage, FCR
[41]	HMBi, HMB and Smartamine M	0.113% 0.204% 0.089%	16	Control, HMBi, HMB and Smartamine M	Milk yield, Milk fat percentage
[42]	RPMet, RPLys	0.11% + 0.05%; 0.122% + 0.045%	30	Control and RPMet + RPLys	Milk yield, Milk fat percentage and Milk lactose percentage, Milk protein percentage
[43]	RPMet, RPLys	0.046% 0.042% 0.066%	84	Control, RPMet and RPLys	Milk yield, Milk fat percentage and FCR
[44]	HMB, HMBi, HMB + HMBi	0.114% 0.166% 0.051% + 0.17%	60	Control, HMB, HMBi and HMB + HMBi	Milk yield, Milk fat percentage and Milk lactose percentage, FCR
[45]	RPMet	0.034%	39	Control and RPMet	Milk yield, Milk fat percentage and Milk lactose percentage, Milk protein percentage
[46]	RPMet	0.0697% 0.1113% 0.0663% 0.1074%	60	Control and RPMet	Milk yield, Milk fat percentage and Milk protein percentage, FCR
[47]	RPMet	0.053% 0.067%	430	Control and RPMet	Milk yield, Milk fat percentage and Milk lactose percentage, Milk protein percentage
[48]	RPMet, RPLys	0.056% 0.085%	4	Control, RPLys, RPMet and RPMet + RPLys	Milk yield, Milk fat percentage and Milk lactose percentage, Milk protein percentage, FCR
[49]	RPMet, RPLys	0.022% 0.058%	39	Control, RPMet and RPLys	Milk yield, Milk fat percentage and Milk lactose percentage, Milk protein percentage, FCR
[50]	HMB	0.15%	60	Control and HMB	Milk yield, Milk fat percentage and Milk lactose percentage, Milk protein percentage, FCR
[51]	RPLys	0.183% 0.185%	40	Control and RPLys	Milk yield, Milk fat percentage and Milk lactose percentage, Milk protein percentage, FCR
[52]	HMBi	0.8%	8	Control and HMBi	Milk yield, Milk fat percentage and Milk lactose percentage, Milk protein percentage
[53]	HMBi	0.082%	30	Control and HMBi	Milk yield, Milk fat percentage and Milk lactose percentage, Milk protein percentage
[54]	RPMet, RPLys	0.10% + 0.06% 0.20% + 0.13%	100	Control and RPMet + RPLys	Milk yield, Milk fat percentage and Milk protein percentage
[55]	RPMet	0.035%	10	Control and RPMet	Milk yield, Milk fat percentage and Milk lactose percentage, Milk protein percentage, FCR
[56]	RPMet	0.036% 0.055% 0.073%	16	Control and RPMet	Milk yield, Milk fat percentage and Milk protein percentage
[57]	RPMet	0.08%	81	Control and RPMet	Milk yield, Milk fat percentage and Milk lactose percentage, Milk protein percentage, FCR

## Data Availability

The datasets used and analyzed during the current study are available from the corresponding authors on reasonable request.

## Supplementary Materials

The following supporting information can be downloaded at: https://www.mdpi.com/article/10.3390/ani16121886/s1, Table S1: Search strategy for identifying relevant studies on PubMed Science direct and Web of Science. Table S2: Quality assessment of studies included in this meta-analysis. Figure S1: Sensitivity analysis of milk yield in Holstein dairy cows. Figure S2: Analysis with study exclusion. Figure S3: Sensitivity analysis of milk fat in Holstein dairy cows. Figure S4: Analysis with study exclusion. Figure S5: Funnel plot of different production performance indicators in Holstein dairy cows. Figure S6: Forest plot of milk yield for different amino acid categories in in Holstein dairy cows. Figure S7: Forest plot of milk fat for different amino acid categories in in Holstein dairy cows. Figure S8: Forest plot of milk lactose for different amino acid categories in Forest plot of milk fat for different amino acid categories in Holstein dairy cows. Figure S9: Forest plot of milk protein for different amino acid categories in Holstein dairy cows. Figure S10: Forest plot of Feed conversion-related traits for different amino acid categories in Holstein dairy cows.
