# Supplementary material for: Effects of Rumen-Protected Lysine and Methionine Supplementation on Lactation Performance in Holstein Dairy Cows: A Meta-Analysis
Source: Animals (Basel). 2026 Jun 18;16(12):1886. doi: 10.3390/ani16121886 (PMC13295602; doi:10.3390/ani16121886)
Supplement: Supplementary file 1 [file animals-16-01886-s001.zip › animals-4340866 - Supplementary.pdf]

Supplementary Figure

Effects of Rumen-Protected Lysine and Methionine Supplementation on Lactation Performance in Dairy Cows: A Meta-Analysis

Authors:

Wenshuo Gao[1], Liyan Jiang[1], Yongling Bao[1], Xiangying Lu[1], Yue hou[1], Lingling Li[1]

Affiliations:

[1] College of Life Science and Technology, Inner Mongolia Normal University, Hohhot 010018, China

***Correspondence author:**

wangjp@imnu.edu.cn **(Jiapeng Wang),** shengtaogao@163.com **(Shengtao Gao)**

Table S1. Search strategy for identifying relevant studies on PubMed Science direct and Web of Science.

Search	Query	Items found
PubMed		
#1	(rumen-protected lysine[Title/Abstract] OR RP lysine[Title/Abstract] OR rumen-escape lysine[Title/Abstract] OR rumen-protected Lys[Title/Abstract] OR RP-Lys[Title/Abstract]) OR rumen-protected methionine[Title/Abstract] OR RP methionine[Title/Abstract] OR rumen-escape methionine[Title/Abstract] OR rumen-protected Met[Title/Abstract] OR RP-Met[Title/Abstract]) OR (rumen-protected lysine[Title/Abstract] OR RP-Lys[Title/Abstract]) AND (rumen-protected methionine[Title/Abstract] OR RP-Met[Title/Abstract])) OR rumen-protected HMB[Title/Abstract]	531
#2	Dairy Cow[Title/Abstract] OR Dairy Cattle[Title/Abstract] OR Dairy Cows[Title/Abstract] OR Lactating Cow[Title/Abstract] OR Lactating Cattle[Title/Abstract] OR Dairy Heifer[Title/Abstract] OR Dairy Calf[Title/Abstract] OR Holstein Cow[Title/Abstract] OR Holstein Cattle[Title/Abstract] OR Holstein Cows[Title/Abstract] OR Holstein-Friesian Cow[Title/Abstract] OR Holstein-Friesian Cattle[Title/Abstract] OR Holstein-Friesian Cows[Title/Abstract] OR Holstein Breed[Title/Abstract] OR Holstein-Friesian Breed[Title/Abstract] OR Lactating Holstein[Title/Abstract]	38760
#3	Milk Yield[Title/Abstract] OR Milk Production[Title/Abstract] OR Daily Milk Yield[Title/Abstract] OR Lactation Milk Yield[Title/Abstract] OR Total Milk Yield[Title/Abstract] OR Milk Output[Title/Abstract] OR Lactational Milk Yield[Title/Abstract] OR Milk Yields[Title/Abstract] OR Production Performance[Title/Abstract] OR Animal Production Performance[Title/Abstract] OR Lactation Performance[Title/Abstract] OR Milk Production Performance[Title/Abstract] OR Lactational Performance[Title/Abstract] OR Lactation Traits[Title/Abstract] OR Milk Protein Percentage[Title/Abstract] OR Milk Protein Percentage[Title/Abstract] OR Milk Protein Concentration[Title/Abstract] OR Milk Crude Protein Percentage[Title/Abstract] OR MP Percentage[Title/Abstract] OR MP	59383

	Percentage[Title/Abstract] OR Milk Protein[Title/Abstract] OR Milk Fat Percentage[Title/Abstract] OR Milk Fat Percentage[Title/Abstract] OR Milk Fat Concentration[Title/Abstract] OR milk fat[Title/Abstract] OR MF Percentage[Title/Abstract] OR Lactose Percentage[Title/Abstract] OR Milk Lactose Percentage[Title/Abstract] OR lactose[Title/Abstract]	
#1 AND #2 AND #3	#1 AND #2 AND #3 AND (("2000/01/01"[Date-Publication]: "2025/01/02"[Date-Publication])) and Article	105
Web of science		
#1	TS=(rumen-protected lysine OR RP-Lys OR rumen-protected methionine OR RP-Met OR rumen-protected methionine analog OR RP-Met analog OR rumen-protected MHA OR rumen-protected HMB OR "2-hydroxy-4-(methylthio)butanoic acid")	1076
#2	TS=(dairy cow OR dairy cattle OR lactating cow OR lactating dairy cow OR Holstein)	113978
#3	TS=(milk yield OR milk production OR productive performance OR milk production performance OR lactation performance OR milk protein percentage OR milk protein percentage OR milk fat percentage OR milk fat percentage OR milk lactose percentage OR milk lactose percentage)	114187
(#1 AND #2 AND#3)	(#1 AND #2 AND#3) AND DOP=(2000-01-01/2025-01-02) and Preprint Citation Index (Exclude – Database) and Article (Document Types)	398

Table S2. Quality assessment of studies included in this meta-analysis.

Study	With-in group Differences[1]	Multiple Reports[2]	Sample Size[3]	Score[4]	Quality
[59]	0	5	2	7	Moderate
[28]	0	5	2	7	Moderate
[29]	0	5	1	6	Low
[51]	0	5	2	7	Moderate
[25]	0	5	2	7	Moderate
[24]	0	5	3	8	Moderate
[18]	0	5	2	7	Moderate
[27]	0	5	5	10	Moderate
[42]	0	5	1	6	Low
[57]	5	5	1	11	Moderate
[47]	0	5	2	7	Moderate
[49]	0	5	1	6	Low
[37]	5	5	2	12	High
[54]	0	5	2	7	Moderate
[56]	0	5	1	6	Low
[22]	0	5	2	7	Moderate
[23]	0	5	3	8	Moderate
[38]	5	5	3	13	High
[40]	0	5	2	7	Moderate
[44]	0	5	3	8	Moderate
[48]	0	5	5	10	Moderate
[19]	0	5	2	7	Moderate
[45]	0	5	2	7	Moderate
[32]	0	5	2	7	Moderate
[34]	0	5	4	9	Moderate
[35]	0	5	4	9	Moderate
[36]	5	5	2	12	High
[60]	0	5	2	7	Moderate
[41]	0	5	1	6	Low
[53]	0	5	1	6	Low
[31]	0	5	2	7	Moderate
[50]	0	5	2	7	Moderate
[46]	5	5	2	12	High
[33]	0	5	2	7	Moderate
[61]	0	5	3	8	Moderate
[52]	0	5	2	7	Moderate
[55]	5	5	4	14	High
[20]	5	5	1	11	Moderate
[30]	0	5	3	8	Moderate
[7]	0	5	3	8	Moderate
[58]	0	5	3	8	Moderate
[43]	5	5	2	12	High

[1] Within-group differences: within-group SD/SE not reported (sub-score = 0); within-group SD/SE reported (sub-score = 5)

[2] Multiple reports: the same dataset presented in different reports (sub-score = 0); no multiple reports (sub-score = 5)

[3] Sample size: >150 (sub-score = 5) ; 100 to 149 (sub-score = 4); 70 to 99 (sub-score = 3); 30 to 69 (sub-score = 2); <30 (sub-score = 1).

[4] Score is the sum of 5 sub-scores. Score of quality:>9 (high); 9 to 20 (medium); ≤7 (low).

Study or Subgroup	Experimental Mean	Experimental SD	Experimental Total	Control Mean	Control SD	Control Total	Weight	Std. Mean Difference IV, Random, 95% CI
**2.1.1 RPLys**								
C.Lee et al.2015	41.5	2.06	2	40.3	2.06	2	0.3%	0.33 [-2.39, 3.05]
D.D.Girma et al.2019	47.1	6.8	17	47.4	6.8	17	1.5%	-0.04 [-0.72, 0.63]
D.D.Girma et al.2019	50.7	6.8	17	47.3	6.8	17	1.5%	0.49 [-0.20, 1.17]
F.Giallongo et al.2016	37.9	2.39	12	38.2	2.39	12	1.3%	-0.12 [-0.92, 0.68]
J.Třináctý et al.2009	32.46	0.88	1	33.33	0.88	1		Not estimable
JIA Ming-jun et al.2010	23.86	0.63	8	23.286	0.85	8	1.1%	0.73 [-0.30, 1.75]
JIA Ming-jun et al.2010	24.189	1	8	23.286	0.85	8	1.1%	0.92 [-0.13, 1.97]
JIA Ming-jun et al.2010	24.634	0.67	8	23.286	0.85	8	1.0%	1.67 [0.48, 2.85]
L.K.Fehlberg et al.2020	40.8	7	18	37.1	7	18	1.5%	0.52 [-0.15, 1.18]
L.K.Fehlberg et al.2020	41.1	7.19	19	37.1	7	18	1.5%	0.55 [-0.11, 1.21]
L.K.Fehlberg et al.2020	40.1	7.38	20	37.1	7	18	1.5%	0.41 [-0.24, 1.05]
Nelson E.Lobos et al.2021	45.4	3.31	8	44.3	3.31	8	1.1%	0.31 [-0.67, 1.30]
P.Melendez et al.2023	32.46	6.48	26	30.74	6.48	26	1.6%	0.26 [-0.28, 0.81]
Pedro Melendez et al.2023	34.9	13.97	26	34.7	3.97	26	1.6%	0.02 [-0.52, 0.56]
V.M.R.Malacco et al.2021	38	2.49	12	38.4	2.49	12	1.3%	-0.16 [-0.96, 0.65]
V.M.R.Malacco et al.2021	37.2	2.49	12	38.4	2.49	12	1.3%	-0.47 [-1.28, 0.35]
Xiaoshi Wei et al.2023	35	1.23	10	33.5	1.23	10	1.2%	1.17 [0.20, 2.13]
Xiaoshi Wei et al.2023	35.2	1.23	10	34.4	1.23	10	1.2%	0.62 [-0.28, 1.53]
**Subtotal (95% CI)**			**234**			**231**	**21.8%**	**0.34 [0.13, 0.55]**
Heterogeneity: Tau² = 0.04; Chi² = 19.78, df = 16 (P = 0.23); I² = 19%								
Test for overall effect: Z = 3.12 (P = 0.002)								
**2.1.2 RPMet**								
F.Giallongo et al.2016	38.5	2.39	12	38.2	2.39	12	1.3%	0.12 [-0.68, 0.92]
G.F.M.Leão et al.2024	42.9	10.39	83	43.1	10.39	83	1.9%	-0.02 [-0.32, 0.29]
HAN Zhao-yu et al.2007	21.79	2.7	4	22.06	2.7	4	0.8%	-0.09 [-1.47, 1.30]
HAN Zhao-yu et al.2007	21.86	2.6	4	22.06	2.7	4	0.8%	-0.07 [-1.45, 1.32]
HAN Zhao-yu et al.2007	22.59	2.3	4	22.06	2.7	4	0.8%	0.18 [-1.21, 1.58]
Hosam H.Titi et al.2013	21.3	0.49	10	21.12	0.49	10	1.2%	0.35 [-0.53, 1.24]
Hosam H.Titi et al.2013	22.62	0.49	10	21.12	0.49	10	0.8%	2.93 [1.59, 4.27]
Hosam H.Titi et al.2013	24.75	0.49	10	22.84	0.49	10	0.7%	3.73 [2.17, 5.29]
Hosam H.Titi et al.2013	25.97	0.49	10	22.84	0.49	10	0.4%	6.12 [3.83, 8.41]
J.Třináctý et al.2009	32.13	0.88	1	33.33	0.88	1		Not estimable
K.Zhao et al.2019	29.4	1.98	2	28.2	1.98	2	0.3%	0.35 [-2.43, 3.12]
M.T.Socha et al.2005	40.9	4.86	14	43.4	4.86	14	1.4%	-0.50 [-1.25, 0.25]
M.T.Socha et al.2005	42.7	4.86	14	42.9	4.86	14	1.4%	-0.04 [-0.78, 0.70]
Mateus Z.Toledo et al.2021	48.7	5.89	217	49.2	5.84	213	2.0%	-0.09 [-0.27, 0.10]
MEHRNAZ ARDALAN et al.2010	32.64	3.07	10	30.71	3.07	10	1.2%	0.60 [-0.30, 1.50]
P.Melendez et al.2023	33.25	6.48	26	30.74	6.48	26	1.6%	0.38 [-0.17, 0.93]
Pedro Melendez et al.2023	36.8	13.97	26	34.7	13.97	26	1.6%	0.15 [-0.40, 0.69]
Tetsuo Tamura et al.2019	40.5	1.6	19	40.4	1.5	20	1.5%	0.06 [-0.56, 0.69]
Yuanxiao Li et al.2022	36.8	0.48	47	36.8	0.49	49	1.8%	0.00 [-0.40, 0.40]
Z.Zhou et al.2016	44.6	7.33	21	41.8	7.16	20	1.6%	0.38 [-0.24, 1.00]
**Subtotal (95% CI)**			**544**			**542**	**23.3%**	**0.39 [0.07, 0.70]**
Heterogeneity: Tau² = 0.28; Chi² = 74.94, df = 18 (P < 0.00001); I² = 76%								
Test for overall effect: Z = 2.43 (P = 0.02)								
**2.1.3 RPLys+RPMet**								
C.Lee et al.2012	36.9	2.56	12	35.2	2.56	12	1.3%	0.64 [-0.18, 1.47]
C.Lee et al.2015	40.2	2.06	2	40.3	2.06	2	0.5%	-0.03 [-1.99, 1.94]
Emrah GÜLGÜN et.al.2022	33.2	2.3	100	31	2.3	100	1.9%	0.95 [0.66, 1.25]
J.Třináctý et al.2009	34.18	0.88	1	33.33	0.88	1		Not estimable
Lu Na et al.2019	41.94	0.13	48	40.47	0.44	48	1.4%	4.49 [3.73, 5.26]
M.T.Socha et al.2005	45.8	4.86	14	43.4	4.86	14	1.4%	0.48 [-0.27, 1.23]
M.T.Socha et al.2005	44	4.86	14	42.9	4.86	14	1.4%	0.22 [-0.52, 0.96]
P.Melendez et al.2023	32.5	6.48	26	30.74	6.48	26	1.6%	0.27 [-0.28, 0.81]
Pedro Melendez et al.2023	36.5	13.97	26	34.7	13.97	26	1.6%	0.13 [-0.42, 0.67]
T.Vanden Bossche et al.2023	30.8	2.16	13	30.6	2.16	13	1.4%	0.09 [-0.68, 0.86]
Xu Yuannian et al.2007	34.75	6.51	50	33.66	5.94	50	1.8%	0.17 [-0.22, 0.57]
Xu Yuannian et al.2007	25.55	5.39	50	24.8	4.48	50	1.8%	0.15 [-0.24, 0.54]
Xu Yuannian et al.2007	37.52	7.41	50	35.43	4.98	50	1.8%	0.33 [-0.07, 0.72]
Xu Yuannian et al.2007	36.5	6.98	50	35.43	6.45	50	1.8%	0.16 [-0.23, 0.55]
ZHAO Ruohan et al.2019	38.5	1.02	10	35.3	1.45	10	0.9%	2.44 [1.23, 3.66]
ZHAO Ruohan et al.2019	37.1	1.08	10	35.3	1.45	10	1.1%	1.35 [0.36, 2.34]
**Subtotal (95% CI)**			**476**			**476**	**21.9%**	**0.76 [0.30, 1.22]**
Heterogeneity: Tau² = 0.68; Chi² = 140.26, df = 14 (P < 0.00001); I² = 90%								
Test for overall effect: Z = 3.24 (P = 0.001)								
**2.1.4 Methionine analog**								
B.C.Benefield et al.2009	36.4	1	4	37.7	1	4	0.7%	-1.13 [-2.72, 0.46]
B.C.Benefield et al.2009	37.2	1	4	37.7	1	4	0.8%	-0.43 [-1.85, 0.98]
B.C.Benefield et al.2009	36.8	1	4	37.7	1	4	0.7%	-0.78 [-2.27, 0.70]
C.Wang et al.2010	28.5	2.52	15	26.5	2.52	15	1.4%	0.77 [0.03, 1.52]
F.X.Amaro et al.2022	39.7	4.65	15	38.4	4.65	15	1.4%	0.27 [-0.45, 0.99]
F.X.Amaro et al.2022	39.6	4.65	15	38.4	4.65	15	1.4%	0.25 [-0.47, 0.97]
F.X.Amaro et al.2022	39.5	4.65	15	38.4	4.65	15	1.4%	0.23 [-0.49, 0.95]
H.Rulquin et al.2006	31.5	2.8	4	31.4	2.8	4	0.8%	0.03 [-1.36, 1.42]
H.Rulquin et al.2006	31.8	2.8	4	31.4	2.8	4	0.8%	0.12 [-1.26, 1.51]
H.Rulquin et al.2006	32	2.8	4	31.4	2.8	4	0.8%	0.19 [-1.21, 1.58]
K.Xia et al.2012	29.54	2.56	10	26.57	2.56	10	1.2%	1.11 [0.15, 2.07]
K.Xia et al.2012	29.75	2.56	10	26.57	2.56	10	1.2%	1.19 [0.22, 2.16]
L.J.Erasmus et al.2013	35.03	3.13	10	35.02	3.13	10	1.3%	0.00 [-0.87, 0.88]
L.J.Erasmus et al.2013	35.17	3.13	10	35.02	3.13	10	1.3%	0.05 [-0.83, 0.92]
M.S.Piepenbrink et al.2004	45	4.4	16	42	4.4	16	1.4%	0.66 [-0.05, 1.38]
M.S.Piepenbrink et al.2004	41.9	4.4	16	42	4.4	16	1.5%	-0.02 [-0.72, 0.67]
N.R.St-Pierre et al.2005	40.7	4.76	15	39.8	4.76	15	1.4%	0.18 [-0.53, 0.90]
N.R.St-Pierre et al.2005	42.3	4.76	15	39.8	4.76	15	1.4%	0.51 [-0.22, 1.24]
N.R.St-Pierre et al.2005	43.2	4.76	15	39.8	4.76	15	1.4%	0.69 [-0.05, 1.44]
PEI Mingcai et al.2023	38.77	1.11	20	37.93	0.71	20	1.5%	0.88 [0.23, 1.54]
R.S.Ordway et al.2009	43.5	4.2	18	43.4	4.43	20	1.5%	0.02 [-0.61, 0.66]
R.S.Ordway et al.2009	42	4.32	19	43.4	4.43	20	1.5%	-0.31 [-0.95, 0.32]
S.E.Räisänen et al.2022	50.1	3.8	6	51.2	3.8	6	1.0%	-0.27 [-1.41, 0.87]
S.J.Whelan et al.2011	26.8	0.55	2	25.2	0.55	2	0.0%	1.66 [-7.95, 11.27]
S.L.Liang et al. 2019	31.91	2.22	12	31.51	2.22	12	1.3%	0.17 [-0.63, 0.98]
S.L.Liang et al. 2019	33.23	2.22	12	31.51	2.22	12	1.3%	0.75 [-0.08, 1.58]
S.L.Liang et al. 2019	31.43	2.22	12	31.51	2.22	12	1.3%	-0.03 [-0.84, 0.77]
YANG Baokui et al.2017	34.32	2.73	12	27.49	4.51	12	1.2%	1.77 [0.80, 2.74]
Z.H.Chen et al.2011	42.1	3.44	14	41.8	3.44	14	0.0%	0.08 [-0.66, 0.83]
**Subtotal (95% CI)**			**314**			**317**	**33.1%**	**0.33 [0.13, 0.52]**
Heterogeneity: Tau² = 0.07; Chi² = 37.15, df = 27 (P = 0.09); I² = 27%								
Test for overall effect: Z = 3.30 (P = 0.0010)								
**Total (95% CI)**			**1568**			**1566**	**100.0%**	**0.44 [0.28, 0.60]**
Heterogeneity: Tau² = 0.32; Chi² = 299.67, df = 78 (P < 0.00001); I² = 74%								
Test for overall effect: Z = 5.45 (P < 0.00001)								
Test for subgroup differences: Chi² = 3.05, df = 3 (P = 0.38), I² = 1.7%								

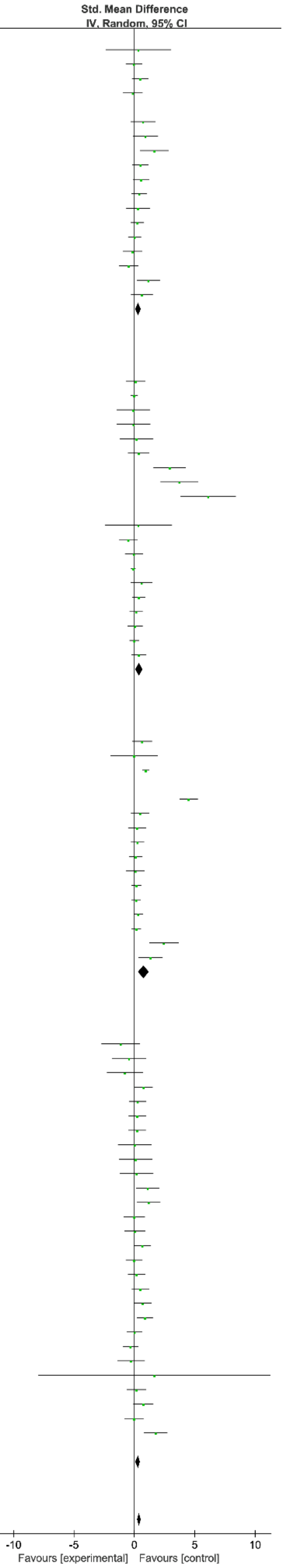

Supplementary Figure S1: Sensitivity analysis of milk yield in Holstein dairy cows.

Study or Subgroup	Experimental Mean	Experimental SD	Experimental Total	Control Mean	Control SD	Control Total	Weight	Std. Mean Difference IV, Random, 95% CI
**2.1.1 RPLys**								
C.Lee et al.2015	41.5	2.06	2	40.3	2.06	2	0.2%	0.33 [-2.39, 3.05]
D.D.Girma et al.2019	47.1	6.8	17	47.4	6.8	17	1.6%	-0.04 [-0.72, 0.63]
D.D.Girma et al.2019	50.7	6.8	17	47.3	6.8	17	1.5%	0.49 [-0.20, 1.17]
F.Giallongo et al.2016	37.9	2.39	12	38.2	2.39	12	1.3%	-0.12 [-0.92, 0.68]
J.Třináctý et al.2009	32.46	0.88	1	33.33	0.88	1		Not estimable
JIA Ming-jun et al.2010	23.86	0.63	8	23.286	0.85	8	1.0%	0.73 [-0.30, 1.75]
JIA Ming-jun et al.2010	24.189	1	8	23.286	0.85	8	1.0%	0.92 [-0.13, 1.97]
JIA Ming-jun et al.2010	24.634	0.67	8	23.286	0.85	8	0.8%	1.67 [0.48, 2.85]
L.K.Fehlberg et al.2020	40.8	7	18	37.1	7	18	1.6%	0.52 [-0.15, 1.18]
L.K.Fehlberg et al.2020	41.1	7.19	19	37.1	7	18	1.6%	0.55 [-0.11, 1.21]
L.K.Fehlberg et al.2020	40.1	7.38	20	37.1	7	18	1.6%	0.41 [-0.24, 1.05]
Nelson E.Lobos et al.2021	45.4	3.31	8	44.3	3.31	8	1.0%	0.31 [-0.67, 1.30]
P.Melendez et al.2023	32.46	6.48	26	30.74	6.48	26	1.8%	0.26 [-0.28, 0.81]
Pedro Melendez et al.2023	34.9	13.97	26	34.7	3.97	26	1.8%	0.02 [-0.52, 0.56]
V.M.R.Malacco et al.2021	38	2.49	12	38.4	2.49	12	1.3%	-0.16 [-0.96, 0.65]
V.M.R.Malacco et al.2021	37.2	2.49	12	38.4	2.49	12	1.3%	-0.47 [-1.28, 0.35]
Xiaoshi Wei et al.2023	35	1.23	10	33.5	1.23	10	1.1%	1.17 [0.20, 2.13]
Xiaoshi Wei et al.2023	35.2	1.23	10	34.4	1.23	10	1.2%	0.62 [-0.28, 1.53]
**Subtotal (95% CI)**			**234**			**231**	**21.6%**	**0.34 [0.13, 0.55]**
Heterogeneity: Tau² = 0.04; Chi² = 19.78, df = 16 (P = 0.23); I² = 19%								
Test for overall effect: Z = 3.12 (P = 0.002)								
**2.1.2 RPMet**								
F.Giallongo et al.2016	38.5	2.39	12	38.2	2.39	12	1.3%	0.12 [-0.68, 0.92]
G.F.M.Leão et al.2024	42.9	10.39	83	43.1	10.39	83	2.4%	-0.02 [-0.32, 0.29]
HAN Zhao-yu et al.2007	21.79	2.7	4	22.06	2.7	4	0.6%	-0.09 [-1.47, 1.30]
HAN Zhao-yu et al.2007	21.86	2.6	4	22.06	2.7	4	0.6%	-0.07 [-1.45, 1.32]
HAN Zhao-yu et al.2007	22.59	2.3	4	22.06	2.7	4	0.6%	0.18 [-1.21, 1.58]
Hosam H.Titi et al.2013	21.3	0.49	10	21.12	0.49	10	1.2%	0.35 [-0.53, 1.24]
Hosam H.Titi et al.2013	22.62	0.49	10	21.12	0.49	10	0.7%	2.93 [1.59, 4.27]
Hosam H.Titi et al.2013	24.75	0.49	10	22.84	0.49	10	0.5%	3.73 [2.17, 5.29]
Hosam H.Titi et al.2013	25.97	0.49	10	22.84	0.49	10	0.3%	6.12 [3.83, 8.41]
J.Třináctý et al.2009	32.13	0.88	1	33.33	0.88	1		Not estimable
K.Zhao et al.2019	29.4	1.98	2	28.2	1.98	2	0.2%	0.35 [-2.43, 3.12]
M.T.Socha et al.2005	40.9	4.86	14	43.4	4.86	14	1.4%	-0.50 [-1.25, 0.25]
M.T.Socha et al.2005	42.7	4.86	14	42.9	4.86	14	1.4%	-0.04 [-0.78, 0.70]
Mateus Z.Toledo et al.2021	48.7	5.89	217	49.2	5.84	213	2.6%	-0.09 [-0.27, 0.10]
MEHRNAZ ARDALAN et al.2010	32.64	3.07	10	30.71	3.07	10	1.2%	0.60 [-0.30, 1.50]
P.Melendez et al.2023	33.25	6.48	26	30.74	6.48	26	1.8%	0.38 [-0.17, 0.93]
Pedro Melendez et al.2023	36.8	13.97	26	34.7	13.97	26	1.8%	0.15 [-0.40, 0.69]
Tetsuo Tamura et al.2019	40.5	1.6	19	40.4	1.5	20	1.6%	0.06 [-0.56, 0.69]
Yuanxiao Li et al.2022	36.8	0.48	47	36.8	0.49	49	2.2%	0.00 [-0.40, 0.40]
Z.Zhou et al.2016	44.6	7.33	21	41.8	7.16	20	1.7%	0.38 [-0.24, 1.00]
**Subtotal (95% CI)**			**544**			**542**	**24.1%**	**0.39 [0.07, 0.70]**
Heterogeneity: Tau² = 0.28; Chi² = 74.94, df = 18 (P < 0.00001); I² = 76%								
Test for overall effect: Z = 2.43 (P = 0.02)								
**2.1.3 RPLys+RPMet**								
C.Lee et al.2012	36.9	2.56	12	35.2	2.56	12	1.3%	0.64 [-0.18, 1.47]
C.Lee et al.2015	40.2	2.06	2	40.3	2.06	2	0.4%	-0.03 [-1.99, 1.94]
Emrah GÜLGÜN et.al.2022	33.2	2.3	100	31	2.3	100	2.4%	0.95 [0.66, 1.25]
J.Třináctý et al.2009	34.18	0.88	1	33.33	0.88	1		Not estimable
Lu Na et al.2019	41.94	0.13	48	40.47	0.44	48	0.0%	4.49 [3.73, 5.26]
M.T.Socha et al.2005	45.8	4.86	14	43.4	4.86	14	1.4%	0.48 [-0.27, 1.23]
M.T.Socha et al.2005	44	4.86	14	42.9	4.86	14	1.4%	0.22 [-0.52, 0.96]
P.Melendez et al.2023	32.5	6.48	26	30.74	6.48	26	1.8%	0.27 [-0.28, 0.81]
Pedro Melendez et al.2023	36.5	13.97	26	34.7	13.97	26	1.8%	0.13 [-0.42, 0.67]
T.Vanden Bossche et al.2023	30.8	2.16	13	30.6	2.16	13	1.4%	0.09 [-0.68, 0.86]
Xu Yuannian et al.2007	34.75	6.51	50	33.66	5.94	50	2.2%	0.17 [-0.22, 0.57]
Xu Yuannian et al.2007	25.55	5.39	50	24.8	4.48	50	2.2%	0.15 [-0.24, 0.54]
Xu Yuannian et al.2007	37.52	7.41	50	35.43	4.98	50	2.2%	0.33 [-0.07, 0.72]
Xu Yuannian et al.2007	36.5	6.98	50	35.43	6.45	50	2.2%	0.16 [-0.23, 0.55]
ZHAO Ruohan et al.2019	38.5	1.02	10	35.3	1.45	10	0.8%	2.44 [1.23, 3.66]
ZHAO Ruohan et al.2019	37.1	1.08	10	35.3	1.45	10	1.0%	1.35 [0.36, 2.34]
**Subtotal (95% CI)**			**428**			**428**	**22.4%**	**0.44 [0.19, 0.69]**
Heterogeneity: Tau² = 0.12; Chi² = 34.80, df = 13 (P = 0.0009); I² = 63%								
Test for overall effect: Z = 3.44 (P = 0.0006)								
**2.1.4 Methionine analog**								
B.C.Benefield et al.2009	36.4	1	4	37.7	1	4	0.5%	-1.13 [-2.72, 0.46]
B.C.Benefield et al.2009	37.2	1	4	37.7	1	4	0.6%	-0.43 [-1.85, 0.98]
B.C.Benefield et al.2009	36.8	1	4	37.7	1	4	0.6%	-0.78 [-2.27, 0.70]
C.Wang et al.2010	28.5	2.52	15	26.5	2.52	15	1.4%	0.77 [0.03, 1.52]
F.X.Amaro et al.2022	39.7	4.65	15	38.4	4.65	15	1.5%	0.27 [-0.45, 0.99]
F.X.Amaro et al.2022	39.6	4.65	15	38.4	4.65	15	1.5%	0.25 [-0.47, 0.97]
F.X.Amaro et al.2022	39.5	4.65	15	38.4	4.65	15	1.5%	0.23 [-0.49, 0.95]
H.Rulquin et al.2006	31.5	2.8	4	31.4	2.8	4	0.6%	0.03 [-1.36, 1.42]
H.Rulquin et al.2006	31.8	2.8	4	31.4	2.8	4	0.6%	0.12 [-1.26, 1.51]
H.Rulquin et al.2006	32	2.8	4	31.4	2.8	4	0.6%	0.19 [-1.21, 1.58]
K.Xia et al.2012	29.54	2.56	10	26.57	2.56	10	1.1%	1.11 [0.15, 2.07]
K.Xia et al.2012	29.75	2.56	10	26.57	2.56	10	1.1%	1.19 [0.22, 2.16]
L.J.Erasmus et al.2013	35.03	3.13	10	35.02	3.13	10	1.2%	0.00 [-0.87, 0.88]
L.J.Erasmus et al.2013	35.17	3.13	10	35.02	3.13	10	1.2%	0.05 [-0.83, 0.92]
M.S.Piepenbrink et al.2004	45	4.4	16	42	4.4	16	1.5%	0.66 [-0.05, 1.38]
M.S.Piepenbrink et al.2004	41.9	4.4	16	42	4.4	16	1.5%	-0.02 [-0.72, 0.67]
N.R.St-Pierre et al.2005	40.7	4.76	15	39.8	4.76	15	1.5%	0.18 [-0.53, 0.90]
N.R.St-Pierre et al.2005	42.3	4.76	15	39.8	4.76	15	1.4%	0.51 [-0.22, 1.24]
N.R.St-Pierre et al.2005	43.2	4.76	15	39.8	4.76	15	1.4%	0.69 [-0.05, 1.44]
PEI Mingcai et al.2023	38.77	1.11	20	37.93	0.71	20	1.6%	0.88 [0.23, 1.54]
R.S.Ordway et al.2009	43.5	4.2	18	43.4	4.43	20	1.6%	0.02 [-0.61, 0.66]
R.S.Ordway et al.2009	42	4.32	19	43.4	4.43	20	1.6%	-0.31 [-0.95, 0.32]
S.E.Räisänen et al.2022	50.1	3.8	6	51.2	3.8	6	0.9%	-0.27 [-1.41, 0.87]
S.J.Whelan et al.2011	26.8	0.55	2	25.2	0.55	2	0.0%	1.66 [-7.95, 11.27]
S.L.Liang et al. 2019	31.91	2.22	12	31.51	2.22	12	1.3%	0.17 [-0.63, 0.98]
S.L.Liang et al. 2019	33.23	2.22	12	31.51	2.22	12	1.3%	0.75 [-0.08, 1.58]
S.L.Liang et al. 2019	31.43	2.22	12	31.51	2.22	12	1.3%	-0.03 [-0.84, 0.77]
YANG Baokui et al.2017	34.32	2.73	12	27.49	4.51	12	1.1%	1.77 [0.80, 2.74]
Z.H.Chen et al.2011	42.1	3.44	14	41.8	3.44	14	0.0%	0.08 [-0.66, 0.83]
**Subtotal (95% CI)**			**314**			**317**	**31.9%**	**0.33 [0.13, 0.52]**
Heterogeneity: Tau² = 0.07; Chi² = 37.15, df = 27 (P = 0.09); I² = 27%								
Test for overall effect: Z = 3.30 (P = 0.0010)								
**Total (95% CI)**			**1520**			**1518**	**100.0%**	**0.36 [0.23, 0.48]**
Heterogeneity: Tau² = 0.15; Chi² = 182.70, df = 77 (P < 0.00001); I² = 58%								
Test for overall effect: Z = 5.52 (P < 0.00001)								
Test for subgroup differences: Chi² = 0.56, df = 3 (P = 0.90), I² = 0%								

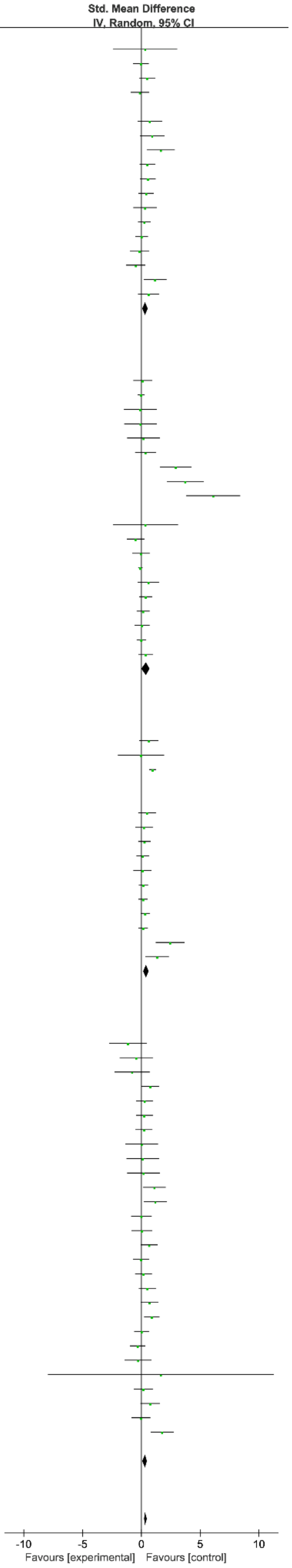

Supplementary Figure S2: Analysis with study exclusion.

Study or Subgroup	Experimental Mean	Experimental SD	Experimental Total	Control Mean	Control SD	Control Total	Weight	Std. Mean Difference IV, Random, 95% CI
**2.2.1 RPLys**								
C.Lee et al.2015	3.3	0.21	2	3.47	0.21	2	0.2%	-0.46 [-3.73, 2.81]
D.D.Girma et al.2019	4.31	1.07	17	3.91	1.07	17	1.5%	0.37 [-0.31, 1.04]
D.D.Girma et al.2019	3.65	1.07	17	4.29	1.07	17	1.5%	-0.58 [-1.27, 0.10]
F.Giallongo et al.2016	3.84	0.43	12	3.72	0.43	12	1.3%	0.27 [-0.54, 1.07]
J.Třináctý et al.2009	3.065	0.2522	1	3.59	0.2522	1		Not estimable
JIA Ming-jun et al.2010	4.431	0.37	8	4.392	0.28	8	1.1%	0.11 [-0.87, 1.09]
JIA Ming-jun et al.2010	4.4	0.42	8	4.392	0.28	8	1.1%	0.02 [-0.96, 1.00]
JIA Ming-jun et al.2010	4.551	0.38	8	4.392	0.28	8	1.1%	0.45 [-0.55, 1.45]
L.K.Fehlberg et al.2020	4.44	0.68	18	4.31	0.68	18	1.5%	0.19 [-0.47, 0.84]
L.K.Fehlberg et al.2020	4.56	0.7	19	4.31	0.68	18	1.5%	0.35 [-0.30, 1.00]
L.K.Fehlberg et al.2020	4.59	0.72	20	4.31	0.68	18	1.5%	0.39 [-0.25, 1.03]
Nelson E.Lobos et al.2021	4.11	0.27	8	4.19	0.27	8	1.1%	-0.28 [-1.27, 0.71]
P.Melendez et al.2023	4.1	0.56	26	4.14	0.56	26	1.7%	-0.07 [-0.61, 0.47]
Pedro Melendez et al.2023	4.49	0.39	26	4.63	0.39	26	1.7%	-0.35 [-0.90, 0.19]
V.M.R.Malacco et al.2021	3.28	0.38	12	3.47	0.38	12	1.3%	-0.48 [-1.30, 0.33]
V.M.R.Malacco et al.2021	3.4	0.38	12	3.47	0.38	12	1.3%	-0.18 [-0.98, 0.62]
Xiaoshi Wei et al.2023	4.09	0.36	10	3.96	0.36	10	1.2%	0.35 [-0.54, 1.23]
Xiaoshi Wei et al.2023	4.01	0.36	10	3.42	0.36	10	1.0%	1.57 [0.54, 2.60]
**Subtotal (95% CI)**			**234**			**231**	**21.3%**	**0.08 [-0.13, 0.30]**
Heterogeneity: Tau² = 0.04; Chi² = 20.59, df = 16 (P = 0.19); I² = 22%								
Test for overall effect: Z = 0.73 (P = 0.46)								
**2.2.2 RPMet**								
F.Giallongo et al.2016	3.8	0.43	12	3.72	0.43	12	1.3%	0.18 [-0.62, 0.98]
G.F.M.Leão et al.2024	4.45	1	83	4.1	1	83	2.0%	0.35 [0.04, 0.65]
HAN Zhao-yu et al.2007	3.99	0.5	4	3.72	0.4	4	0.7%	0.52 [-0.91, 1.95]
HAN Zhao-yu et al.2007	3.73	0.4	4	3.72	0.4	4	0.7%	0.02 [-1.36, 1.41]
HAN Zhao-yu et al.2007	3.86	0.4	4	3.72	0.4	4	0.7%	0.30 [-1.10, 1.71]
Hosam H.Titi et al.2013	3.49	0.05	10	3.42	0.05	10	1.1%	1.34 [0.35, 2.33]
Hosam H.Titi et al.2013	3.61	0.05	10	3.42	0.05	10	0.6%	3.64 [2.11, 5.17]
Hosam H.Titi et al.2013	3.54	0.05	10	3.48	0.05	10	1.1%	1.15 [0.19, 2.11]
Hosam H.Titi et al.2013	3.55	0.05	10	3.48	0.05	10	1.1%	1.34 [0.35, 2.33]
J.Třináctý et al.2009	3.191	0.2522	1	3.59	0.2522	1		Not estimable
K.Zhao et al.2019	4.16	0.3	2	3.95	0.3	2	0.2%	0.40 [-2.59, 3.39]
M.T.Socha et al.2005	3.88	0.22	14	3.62	0.22	14	1.3%	1.15 [0.34, 1.96]
M.T.Socha et al.2005	3.63	0.22	14	3.69	0.22	14	1.4%	-0.26 [-1.01, 0.48]
Mateus Z.Toledo et al.2021	3.87	0.44	217	3.77	0.44	213	2.1%	0.23 [0.04, 0.42]
MEHRNAZ ARDALAN et al.2010	3.3	0.09	10	3.29	0.09	10	1.2%	0.11 [-0.77, 0.98]
P.Melendez et al.2023	4.26	0.56	26	4.14	0.56	26	1.7%	0.21 [-0.33, 0.76]
Pedro Melendez et al.2023	4.52	0.39	26	4.63	0.39	26	1.7%	-0.28 [-0.82, 0.27]
Tetsuo Tamura et al.2019	3.74	0.19	19	3.61	0.18	20	1.5%	0.69 [0.04, 1.34]
Yuanxiao Li et al.2022	3.8	0.21	47	3.49	0.21	49	1.8%	1.46 [1.01, 1.92]
Z.Zhou et al.2016	3.75	0.69	21	3.61	0.67	20	1.6%	0.20 [-0.41, 0.82]
**Subtotal (95% CI)**			**544**			**542**	**23.6%**	**0.57 [0.28, 0.87]**
Heterogeneity: Tau² = 0.24; Chi² = 65.86, df = 18 (P < 0.00001); I² = 73%								
Test for overall effect: Z = 3.84 (P = 0.0001)								
**2.2.3 RPLys+RPMet**								
C.Lee et al.2012	3.32	0.41	12	3.51	0.41	12	1.3%	-0.45 [-1.26, 0.36]
C.Lee et al.2015	3.41	0.21	2	3.47	0.21	2	0.4%	-0.16 [-2.33, 2.00]
Emrah GÜLGÜN et.al.2022	3.87	0.2	100	3.77	0.2	100	2.0%	0.50 [0.22, 0.78]
J.Třináctý et al.2009	3.372	0.2522	1	3.59	0.2522	1		Not estimable
Lu Na et al.2019	4.26	0.13	48	4.26	0.1	48	1.9%	0.00 [-0.40, 0.40]
M.T.Socha et al.2005	3.74	0.22	14	3.69	0.22	14	1.4%	0.22 [-0.52, 0.96]
M.T.Socha et al.2005	3.65	0.22	14	3.62	0.22	14	1.4%	0.13 [-0.61, 0.87]
P.Melendez et al.2023	4.05	0.56	26	4.14	0.56	26	1.7%	-0.16 [-0.70, 0.39]
Pedro Melendez et al.2023	4.68	0.39	26	4.63	0.39	26	1.7%	0.13 [-0.42, 0.67]
T.Vanden Bossche et al.2023	4.35	0.29	13	4.34	0.29	13	1.3%	0.03 [-0.74, 0.80]
Xu Yuannian et al.2007	3.36	0.31	50	3.31	0.46	50	1.9%	0.13 [-0.27, 0.52]
Xu Yuannian et al.2007	3.48	0.77	50	3.31	0.46	50	1.9%	0.27 [-0.13, 0.66]
Xu Yuannian et al.2007	3.35	0.34	50	3.24	0.31	50	1.9%	0.34 [-0.06, 0.73]
Xu Yuannian et al.2007	3.13	0.26	50	3.57	0.47	50	1.8%	-1.15 [-1.57, -0.73]
ZHAO Ruohan et al.2019	3.44	0.15	10	3.89	0.12	10	0.7%	-3.17 [-4.58, -1.77]
ZHAO Ruohan et al.2019	3.59	0.16	10	3.89	0.12	10	0.9%	-2.03 [-3.16, -0.91]
**Subtotal (95% CI)**			**476**			**476**	**22.0%**	**-0.21 [-0.55, 0.14]**
Heterogeneity: Tau² = 0.33; Chi² = 79.40, df = 14 (P < 0.00001); I² = 82%								
Test for overall effect: Z = 1.18 (P = 0.24)								
**2.2.4 Methionine analog**								
B.C.Benefield et al.2009	3.3	0.08	4	3.21	0.08	4	0.6%	0.98 [-0.56, 2.52]
B.C.Benefield et al.2009	3.35	0.08	4	3.21	0.08	4	0.5%	1.52 [-0.21, 3.26]
B.C.Benefield et al.2009	3.33	0.08	4	3.21	0.08	4	0.6%	1.30 [-0.35, 2.96]
C.Wang et al.2010	3.95	0.19	15	3.6	0.19	15	1.2%	1.79 [0.93, 2.66]
F.X.Amaro et al.2022	3.54	0.54	15	3.54	0.54	15	1.4%	0.00 [-0.72, 0.72]
F.X.Amaro et al.2022	3.31	0.54	15	3.54	0.54	15	1.4%	-0.41 [-1.14, 0.31]
F.X.Amaro et al.2022	3.3	0.54	15	3.54	0.54	15	1.4%	-0.43 [-1.16, 0.29]
H.Rulquin et al.2006	4.2	0.3	4	4.16	0.3	4	0.7%	0.12 [-1.27, 1.50]
H.Rulquin et al.2006	4.26	0.3	4	4.16	0.3	4	0.7%	0.29 [-1.11, 1.69]
H.Rulquin et al.2006	4.16	0.3	4	4.16	0.3	4	0.7%	0.00 [-1.39, 1.39]
K.Xia et al.2012	3.78	0.149	10	3.69	0.149	10	1.2%	0.58 [-0.32, 1.48]
K.Xia et al.2012	3.89	0.149	10	3.69	0.149	10	1.1%	1.29 [0.30, 2.27]
L.J.Erasmus et al.2013	3.93	0.44	10	3.84	0.44	10	1.2%	0.20 [-0.68, 1.08]
L.J.Erasmus et al.2013	4.27	0.44	10	3.84	0.44	10	1.1%	0.94 [0.00, 1.87]
M.S.Piepenbrink et al.2004	4	0.36	16	4.2	0.36	16	1.4%	-0.54 [-1.25, 0.17]
M.S.Piepenbrink et al.2004	4.08	0.36	16	4.2	0.36	16	1.4%	-0.32 [-1.02, 0.37]
N.R.St-Pierre et al.2005	3.76	0.43	15	3.61	0.43	15	1.4%	0.34 [-0.38, 1.06]
N.R.St-Pierre et al.2005	3.82	0.43	15	3.61	0.43	15	1.4%	0.48 [-0.25, 1.20]
N.R.St-Pierre et al.2005	3.86	0.43	15	3.61	0.43	15	1.4%	0.57 [-0.17, 1.30]
PEI Mingcai et al.2023	3.88	0.05	20	3.8	0.05	20	1.4%	1.57 [0.85, 2.29]
R.S.Ordway et al.2009	3.65	0.31	18	3.65	0.33	20	1.5%	0.00 [-0.64, 0.64]
R.S.Ordway et al.2009	3.68	0.32	19	3.65	0.33	20	1.5%	0.09 [-0.54, 0.72]
S.E.Räisänen et al.2022	3.47	0.4	6	3.77	0.4	6	0.9%	-0.69 [-1.87, 0.49]
S.J.Whelan et al.2011	3.79	0.13	2	3.76	0.13	2	0.4%	0.13 [-1.97, 2.23]
S.L.Liang et al. 2019	3.98	0.45	12	4	0.45	12	1.3%	-0.04 [-0.84, 0.76]
S.L.Liang et al. 2019	4.05	0.45	12	4	0.45	12	1.3%	0.11 [-0.69, 0.91]
S.L.Liang et al. 2019	3.95	0.45	12	4	0.45	12	1.3%	-0.11 [-0.91, 0.69]
YANG Baokui et al.2017	3.84	0.08	12	3.73	0.04	12	1.1%	1.68 [0.73, 2.63]
Z.H.Chen et al.2011	3.93	0.41	14	3.52	0.41	14	1.3%	0.97 [0.18, 1.76]
**Subtotal (95% CI)**			**328**			**331**	**33.0%**	**0.38 [0.12, 0.64]**
Heterogeneity: Tau² = 0.28; Chi² = 68.04, df = 28 (P < 0.0001); I² = 59%								
Test for overall effect: Z = 2.86 (P = 0.004)								
**Total (95% CI)**			**1582**			**1580**	**100.0%**	**0.24 [0.10, 0.38]**
Heterogeneity: Tau² = 0.25; Chi² = 255.64, df = 79 (P < 0.00001); I² = 69%								
Test for overall effect: Z = 3.28 (P = 0.001)								
Test for subgroup differences: Chi² = 14.65, df = 3 (P = 0.002), I² = 79.5%								

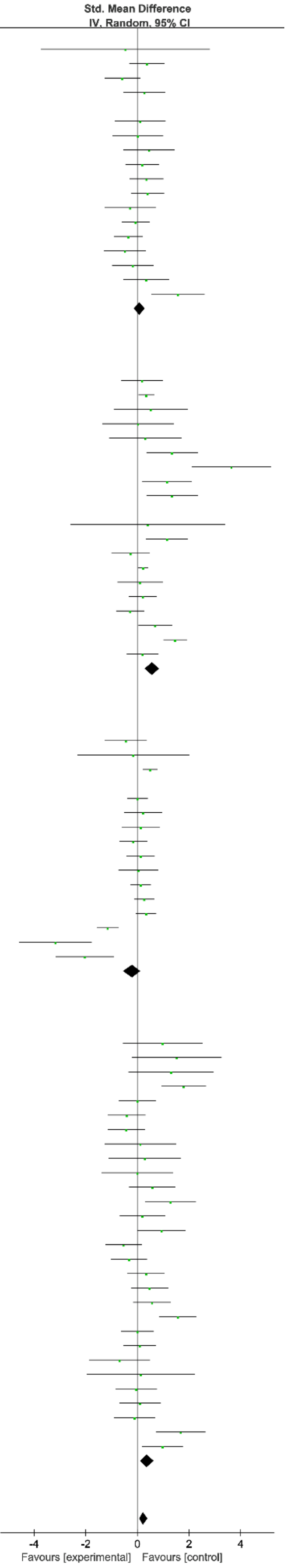

Supplementary Figure S3: Sensitivity analysis of milk fat in Holstein dairy cows.

	Experimental			Control				Std. Mean Difference
Study or Subgroup	Mean	SD	Total	Mean	SD	Total	Weight	IV, Random, 95% CI
**2.2.1 RPLys**								
C.Lee et al.2015	3.3	0.21	2	3.47	0.21	2	0.2%	-0.46 [-3.73, 2.81]
D.D.Girma et al.2019	4.31	1.07	17	3.91	1.07	17	1.5%	0.37 [-0.31, 1.04]
D.D.Girma et al.2019	3.65	1.07	17	4.29	1.07	17	1.5%	-0.58 [-1.27, 0.10]
F.Giallongo et al.2016	3.84	0.43	12	3.72	0.43	12	1.3%	0.27 [-0.54, 1.07]
J.Třináctý et al.2009	3.065	0.2522	1	3.59	0.2522	1		Not estimable
JIA Ming-jun et al.2010	4.431	0.37	8	4.392	0.28	8	1.1%	0.11 [-0.87, 1.09]
JIA Ming-jun et al.2010	4.4	0.42	8	4.392	0.28	8	1.1%	0.02 [-0.96, 1.00]
JIA Ming-jun et al.2010	4.551	0.38	8	4.392	0.28	8	1.1%	0.45 [-0.55, 1.45]
L.K.Fehlberg et al.2020	4.44	0.68	18	4.31	0.68	18	1.5%	0.19 [-0.47, 0.84]
L.K.Fehlberg et al.2020	4.56	0.7	19	4.31	0.68	18	1.5%	0.35 [-0.30, 1.00]
L.K.Fehlberg et al.2020	4.59	0.72	20	4.31	0.68	18	1.5%	0.39 [-0.25, 1.03]
Nelson E.Lobos et al.2021	4.11	0.27	8	4.19	0.27	8	1.1%	-0.28 [-1.27, 0.71]
P.Melendez et al.2023	4.1	0.56	26	4.14	0.56	26	1.7%	-0.07 [-0.61, 0.47]
Pedro Melendez et al.2023	4.49	0.39	26	4.63	0.39	26	1.7%	-0.35 [-0.90, 0.19]
V.M.R.Malacco et al.2021	3.28	0.38	12	3.47	0.38	12	1.3%	-0.48 [-1.30, 0.33]
V.M.R.Malacco et al.2021	3.4	0.38	12	3.47	0.38	12	1.3%	-0.18 [-0.98, 0.62]
Xiaoshi Wei et al.2023	4.09	0.36	10	3.96	0.36	10	1.2%	0.35 [-0.54, 1.23]
Xiaoshi Wei et al.2023	4.01	0.36	10	3.42	0.36	10	0.0%	1.57 [0.54, 2.60]
**Subtotal (95% CI)**			**224**			**221**	**20.5%**	**0.02 [-0.17, 0.21]**
Heterogeneity: Tau² = 0.00; Chi² = 12.16, df = 15 (P = 0.67); I² = 0%								
Test for overall effect: Z = 0.18 (P = 0.86)								
**2.2.2 RPMet**								
F.Giallongo et al.2016	3.8	0.43	12	3.72	0.43	12	1.3%	0.18 [-0.62, 0.98]
G.F.M.Leão et al.2024	4.45	1	83	4.1	1	83	2.0%	0.35 [0.04, 0.65]
HAN Zhao-yu et al.2007	3.99	0.5	4	3.72	0.4	4	0.7%	0.52 [-0.91, 1.95]
HAN Zhao-yu et al.2007	3.73	0.4	4	3.72	0.4	4	0.7%	0.02 [-1.36, 1.41]
HAN Zhao-yu et al.2007	3.86	0.4	4	3.72	0.4	4	0.7%	0.30 [-1.10, 1.71]
Hosam H.Titi et al.2013	3.49	0.05	10	3.42	0.05	10	1.1%	1.34 [0.35, 2.33]
Hosam H.Titi et al.2013	3.61	0.05	10	3.42	0.05	10	0.6%	3.64 [2.11, 5.17]
Hosam H.Titi et al.2013	3.54	0.05	10	3.48	0.05	10	1.1%	1.15 [0.19, 2.11]
Hosam H.Titi et al.2013	3.55	0.05	10	3.48	0.05	10	1.1%	1.34 [0.35, 2.33]
J.Třináctý et al.2009	3.191	0.2522	1	3.59	0.2522	1		Not estimable
K.Zhao et al.2019	4.16	0.3	2	3.95	0.3	2	0.2%	0.40 [-2.59, 3.39]
M.T.Socha et al.2005	3.88	0.22	14	3.62	0.22	14	1.3%	1.15 [0.34, 1.96]
M.T.Socha et al.2005	3.63	0.22	14	3.69	0.22	14	1.4%	-0.26 [-1.01, 0.48]
Mateus Z.Toledo et al.2021	3.87	0.44	217	3.77	0.44	213	2.1%	0.23 [0.04, 0.42]
MEHRNAZ ARDALAN et al.2010	3.3	0.09	10	3.29	0.09	10	1.2%	0.11 [-0.77, 0.98]
P.Melendez et al.2023	4.26	0.56	26	4.14	0.56	26	1.7%	0.21 [-0.33, 0.76]
Pedro Melendez et al.2023	4.52	0.39	26	4.63	0.39	26	1.7%	-0.28 [-0.82, 0.27]
Tetsuo Tamura et al.2019	3.74	0.19	19	3.61	0.18	20	1.5%	0.69 [0.04, 1.34]
Yuanxiao Li et al.2022	3.8	0.21	47	3.49	0.21	49	1.8%	1.46 [1.01, 1.92]
Z.Zhou et al.2016	3.75	0.69	21	3.61	0.67	20	1.6%	0.20 [-0.41, 0.82]
**Subtotal (95% CI)**			**544**			**542**	**23.9%**	**0.57 [0.28, 0.87]**
Heterogeneity: Tau² = 0.24; Chi² = 65.86, df = 18 (P < 0.00001); I² = 73%								
Test for overall effect: Z = 3.84 (P = 0.0001)								
**2.2.3 RPLys+RPMet**								
C.Lee et al.2012	3.32	0.41	12	3.51	0.41	12	1.3%	-0.45 [-1.26, 0.36]
C.Lee et al.2015	3.41	0.21	2	3.47	0.21	2	0.4%	-0.16 [-2.33, 2.00]
Emrah GÜLGÜN et.al.2022	3.87	0.2	100	3.77	0.2	100	2.0%	0.50 [0.22, 0.78]
J.Třináctý et al.2009	3.372	0.2522	1	3.59	0.2522	1		Not estimable
Lu Na et al.2019	4.26	0.13	48	4.26	0.1	48	1.9%	0.00 [-0.40, 0.40]
M.T.Socha et al.2005	3.74	0.22	14	3.69	0.22	14	1.4%	0.22 [-0.52, 0.96]
M.T.Socha et al.2005	3.65	0.22	14	3.62	0.22	14	1.4%	0.13 [-0.61, 0.87]
P.Melendez et al.2023	4.05	0.56	26	4.14	0.56	26	1.7%	-0.16 [-0.70, 0.39]
Pedro Melendez et al.2023	4.68	0.39	26	4.63	0.39	26	1.7%	0.13 [-0.42, 0.67]
T.Vanden Bossche et al.2023	4.35	0.29	13	4.34	0.29	13	1.4%	0.03 [-0.74, 0.80]
Xu Yuannian et al.2007	3.36	0.31	50	3.31	0.46	50	1.9%	0.13 [-0.27, 0.52]
Xu Yuannian et al.2007	3.48	0.77	50	3.31	0.46	50	1.9%	0.27 [-0.13, 0.66]
Xu Yuannian et al.2007	3.35	0.34	50	3.24	0.31	50	1.9%	0.34 [-0.06, 0.73]
Xu Yuannian et al.2007	3.13	0.26	50	3.57	0.47	50	1.9%	-1.15 [-1.57, -0.73]
ZHAO Ruohan et al.2019	3.44	0.15	10	3.89	0.12	10	0.7%	-3.17 [-4.58, -1.77]
ZHAO Ruohan et al.2019	3.59	0.16	10	3.89	0.12	10	0.9%	-2.03 [-3.16, -0.91]
**Subtotal (95% CI)**			**476**			**476**	**22.3%**	**-0.21 [-0.55, 0.14]**
Heterogeneity: Tau² = 0.33; Chi² = 79.40, df = 14 (P < 0.00001); I² = 82%								
Test for overall effect: Z = 1.18 (P = 0.24)								
**2.2.4 Methionine analog**								
B.C.Benefield et al.2009	3.3	0.08	4	3.21	0.08	4	0.6%	0.98 [-0.56, 2.52]
B.C.Benefield et al.2009	3.35	0.08	4	3.21	0.08	4	0.5%	1.52 [-0.21, 3.26]
B.C.Benefield et al.2009	3.33	0.08	4	3.21	0.08	4	0.6%	1.30 [-0.35, 2.96]
C.Wang et al.2010	3.95	0.19	15	3.6	0.19	15	1.2%	1.79 [0.93, 2.66]
F.X.Amaro et al.2022	3.54	0.54	15	3.54	0.54	15	1.4%	0.00 [-0.72, 0.72]
F.X.Amaro et al.2022	3.31	0.54	15	3.54	0.54	15	1.4%	-0.41 [-1.14, 0.31]
F.X.Amaro et al.2022	3.3	0.54	15	3.54	0.54	15	1.4%	-0.43 [-1.16, 0.29]
H.Rulquin et al.2006	4.2	0.3	4	4.16	0.3	4	0.7%	0.12 [-1.27, 1.50]
H.Rulquin et al.2006	4.26	0.3	4	4.16	0.3	4	0.7%	0.29 [-1.11, 1.69]
H.Rulquin et al.2006	4.16	0.3	4	4.16	0.3	4	0.7%	0.00 [-1.39, 1.39]
K.Xia et al.2012	3.78	0.149	10	3.69	0.149	10	1.2%	0.58 [-0.32, 1.48]
K.Xia et al.2012	3.89	0.149	10	3.69	0.149	10	1.1%	1.29 [0.30, 2.27]
L.J.Erasmus et al.2013	3.93	0.44	10	3.84	0.44	10	1.2%	0.20 [-0.68, 1.08]
L.J.Erasmus et al.2013	4.27	0.44	10	3.84	0.44	10	1.1%	0.94 [0.00, 1.87]
M.S.Piepenbrink et al.2004	4	0.36	16	4.2	0.36	16	1.4%	-0.54 [-1.25, 0.17]
M.S.Piepenbrink et al.2004	4.08	0.36	16	4.2	0.36	16	1.5%	-0.32 [-1.02, 0.37]
N.R.St-Pierre et al.2005	3.76	0.43	15	3.61	0.43	15	1.4%	0.34 [-0.38, 1.06]
N.R.St-Pierre et al.2005	3.82	0.43	15	3.61	0.43	15	1.4%	0.48 [-0.25, 1.20]
N.R.St-Pierre et al.2005	3.86	0.43	15	3.61	0.43	15	1.4%	0.57 [-0.17, 1.30]
PEI Mingcai et al.2023	3.88	0.05	20	3.8	0.05	20	1.4%	1.57 [0.85, 2.29]
R.S.Ordway et al.2009	3.65	0.31	18	3.65	0.33	20	1.5%	0.00 [-0.64, 0.64]
R.S.Ordway et al.2009	3.68	0.32	19	3.65	0.33	20	1.6%	0.09 [-0.54, 0.72]
S.E.Räisänen et al.2022	3.47	0.4	6	3.77	0.4	6	0.9%	-0.69 [-1.87, 0.49]
S.J.Whelan et al.2011	3.79	0.13	2	3.76	0.13	2	0.4%	0.13 [-1.97, 2.23]
S.L.Liang et al. 2019	3.98	0.45	12	4	0.45	12	1.3%	-0.04 [-0.84, 0.76]
S.L.Liang et al. 2019	4.05	0.45	12	4	0.45	12	1.3%	0.11 [-0.69, 0.91]
S.L.Liang et al. 2019	3.95	0.45	12	4	0.45	12	1.3%	-0.11 [-0.91, 0.69]
YANG Baokui et al.2017	3.84	0.08	12	3.73	0.04	12	1.1%	1.68 [0.73, 2.63]
Z.H.Chen et al.2011	3.93	0.41	14	3.52	0.41	14	1.3%	0.97 [0.18, 1.76]
**Subtotal (95% CI)**			**328**			**331**	**33.3%**	**0.38 [0.12, 0.64]**
Heterogeneity: Tau² = 0.28; Chi² = 68.04, df = 28 (P < 0.0001); I² = 59%								
Test for overall effect: Z = 2.86 (P = 0.004)								
**Total (95% CI)**			**1572**			**1570**	**100.0%**	**0.23 [0.08, 0.37]**
Heterogeneity: Tau² = 0.24; Chi² = 249.03, df = 78 (P < 0.00001); I² = 69%								
Test for overall effect: Z = 3.10 (P = 0.002)								
Test for subgroup differences: Chi² = 16.99, df = 3 (P = 0.0007), I² = 82.3%								

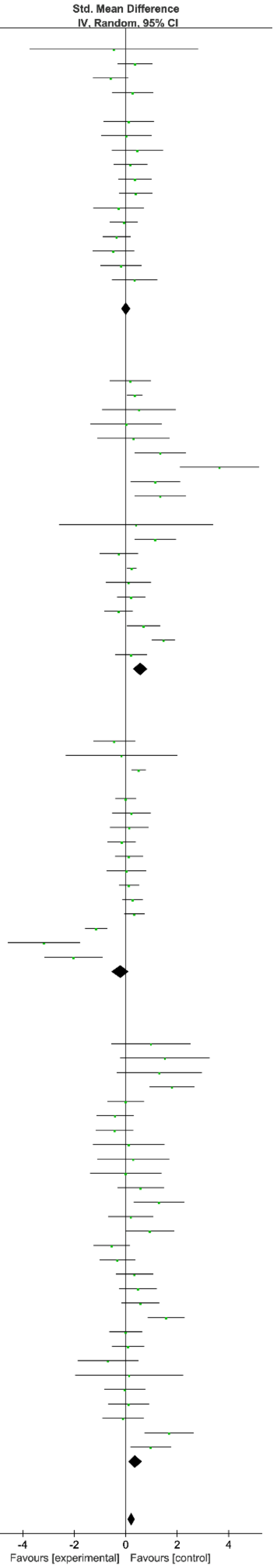

Supplementary Figure S4: Analysis with study exclusion.

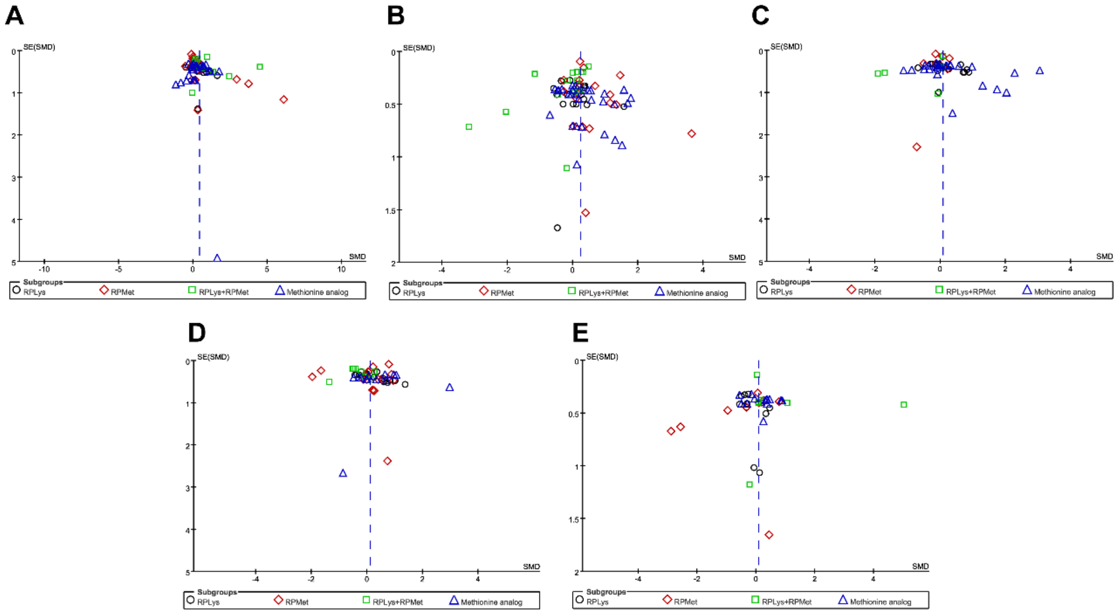

Supplementary Figure S5: Funnel plot of different production performance indicators in Holstein dairy cows. (A) Milk yield. (B) Milk fat. (C) Milk lactose. (D) Milk protein. (E) Feed conversion-related traits.

Study or Subgroup	Experimental Mean	Experimental SD	Experimental Total	Control Mean	Control SD	Control Total	Weight	Std. Mean Difference IV, Random, 95% CI
**2.1.1 RPLys**								
C.Lee et al.2015	41.5	2.06	2	40.3	2.06	2	0.3%	0.33 [-2.39, 3.05]
D.D.Girma et al.2019	47.1	6.8	17	47.4	6.8	17	1.5%	-0.04 [-0.72, 0.63]
D.D.Girma et al.2019	50.7	6.8	17	47.3	6.8	17	1.5%	0.49 [-0.20, 1.17]
F.Giallongo et al.2016	37.9	2.39	12	38.2	2.39	12	1.3%	-0.12 [-0.92, 0.68]
J.Třináctý et al.2009	32.46	0.88	1	33.33	0.88	1		Not estimable
JIA Ming-jun et al.2010	23.86	0.63	8	23.286	0.85	8	1.1%	0.73 [-0.30, 1.75]
JIA Ming-jun et al.2010	24.189	1	8	23.286	0.85	8	1.1%	0.92 [-0.13, 1.97]
JIA Ming-jun et al.2010	24.634	0.67	8	23.286	0.85	8	1.0%	1.67 [0.48, 2.85]
L.K.Fehlberg et al.2020	40.8	7	18	37.1	7	18	1.5%	0.52 [-0.15, 1.18]
L.K.Fehlberg et al.2020	41.1	7.19	19	37.1	7	18	1.5%	0.55 [-0.11, 1.21]
L.K.Fehlberg et al.2020	40.1	7.38	20	37.1	7	18	1.5%	0.41 [-0.24, 1.05]
Nelson E.Lobos et al.2021	45.4	3.31	8	44.3	3.31	8	1.1%	0.31 [-0.67, 1.30]
P.Melendez et al.2023	32.46	6.48	26	30.74	6.48	26	1.6%	0.26 [-0.28, 0.81]
Pedro Melendez et al.2023	34.9	13.97	26	34.7	3.97	26	1.6%	0.02 [-0.52, 0.56]
V.M.R.Malacco et al.2021	38	2.49	12	38.4	2.49	12	1.3%	-0.16 [-0.96, 0.65]
V.M.R.Malacco et al.2021	37.2	2.49	12	38.4	2.49	12	1.3%	-0.47 [-1.28, 0.35]
Xiaoshi Wei et al.2023	35	1.23	10	33.5	1.23	10	1.2%	1.17 [0.20, 2.13]
Xiaoshi Wei et al.2023	35.2	1.23	10	34.4	1.23	10	1.2%	0.62 [-0.28, 1.53]
**Subtotal (95% CI)**			**234**			**231**	**21.8%**	**0.34 [0.13, 0.55]**
Heterogeneity: Tau² = 0.04; Chi² = 19.78, df = 16 (P = 0.23); I² = 19%								
Test for overall effect: Z = 3.12 (P = 0.002)								
**2.1.2 RPMet**								
F.Giallongo et al.2016	38.5	2.39	12	38.2	2.39	12	1.3%	0.12 [-0.68, 0.92]
G.F.M.Leão et al.2024	42.9	10.39	83	43.1	10.39	83	1.9%	-0.02 [-0.32, 0.29]
HAN Zhao-yu et al.2007	21.79	2.7	4	22.06	2.7	4	0.8%	-0.09 [-1.47, 1.30]
HAN Zhao-yu et al.2007	21.86	2.6	4	22.06	2.7	4	0.8%	-0.07 [-1.45, 1.32]
HAN Zhao-yu et al.2007	22.59	2.3	4	22.06	2.7	4	0.8%	0.18 [-1.21, 1.58]
Hosam H.Titi et al.2013	21.3	0.49	10	21.12	0.49	10	1.2%	0.35 [-0.53, 1.24]
Hosam H.Titi et al.2013	22.62	0.49	10	21.12	0.49	10	0.8%	2.93 [1.59, 4.27]
Hosam H.Titi et al.2013	24.75	0.49	10	22.84	0.49	10	0.7%	3.73 [2.17, 5.29]
Hosam H.Titi et al.2013	25.97	0.49	10	22.84	0.49	10	0.4%	6.12 [3.83, 8.41]
J.Třináctý et al.2009	32.13	0.88	1	33.33	0.88	1		Not estimable
K.Zhao et al.2019	29.4	1.98	2	28.2	1.98	2	0.3%	0.35 [-2.43, 3.12]
M.T.Socha et al.2005	40.9	4.86	14	43.4	4.86	14	1.4%	-0.50 [-1.25, 0.25]
M.T.Socha et al.2005	42.7	4.86	14	42.9	4.86	14	1.4%	-0.04 [-0.78, 0.70]
Mateus Z.Toledo et al.2021	48.7	5.89	217	49.2	5.84	213	2.0%	-0.09 [-0.27, 0.10]
MEHRNAZ ARDALAN et al.2010	32.64	3.07	10	30.71	3.07	10	1.2%	0.60 [-0.30, 1.50]
P.Melendez et al.2023	33.25	6.48	26	30.74	6.48	26	1.6%	0.38 [-0.17, 0.93]
Pedro Melendez et al.2023	36.8	13.97	26	34.7	13.97	26	1.6%	0.15 [-0.40, 0.69]
Tetsuo Tamura et al.2019	40.5	1.6	19	40.4	1.5	20	1.5%	0.06 [-0.56, 0.69]
Yuanxiao Li et al.2022	36.8	0.48	47	36.8	0.49	49	1.8%	0.00 [-0.40, 0.40]
Z.Zhou et al.2016	44.6	7.33	21	41.8	7.16	20	1.6%	0.38 [-0.24, 1.00]
**Subtotal (95% CI)**			**544**			**542**	**23.3%**	**0.39 [0.07, 0.70]**
Heterogeneity: Tau² = 0.28; Chi² = 74.94, df = 18 (P < 0.00001); I² = 76%								
Test for overall effect: Z = 2.43 (P = 0.02)								
**2.1.3 RPLys+RPMet**								
C.Lee et al.2012	36.9	2.56	12	35.2	2.56	12	1.3%	0.64 [-0.18, 1.47]
C.Lee et al.2015	40.2	2.06	2	40.3	2.06	2	0.5%	-0.03 [-1.99, 1.94]
Emrah GÜLGÜN et.al.2022	33.2	2.3	100	31	2.3	100	1.9%	0.95 [0.66, 1.25]
J.Třináctý et al.2009	34.18	0.88	1	33.33	0.88	1		Not estimable
Lu Na et al.2019	41.94	0.13	48	40.47	0.44	48	1.4%	4.49 [3.73, 5.26]
M.T.Socha et al.2005	45.8	4.86	14	43.4	4.86	14	1.4%	0.48 [-0.27, 1.23]
M.T.Socha et al.2005	44	4.86	14	42.9	4.86	14	1.4%	0.22 [-0.52, 0.96]
P.Melendez et al.2023	32.5	6.48	26	30.74	6.48	26	1.6%	0.27 [-0.28, 0.81]
Pedro Melendez et al.2023	36.5	13.97	26	34.7	13.97	26	1.6%	0.13 [-0.42, 0.67]
T.Vanden Bossche et al.2023	30.8	2.16	13	30.6	2.16	13	1.4%	0.09 [-0.68, 0.86]
Xu Yuannian et al.2007	34.75	6.51	50	33.66	5.94	50	1.8%	0.17 [-0.22, 0.57]
Xu Yuannian et al.2007	25.55	5.39	50	24.8	4.48	50	1.8%	0.15 [-0.24, 0.54]
Xu Yuannian et al.2007	37.52	7.41	50	35.43	4.98	50	1.8%	0.33 [-0.07, 0.72]
Xu Yuannian et al.2007	36.5	6.98	50	35.43	6.45	50	1.8%	0.16 [-0.23, 0.55]
ZHAO Ruohan et al.2019	38.5	1.02	10	35.3	1.45	10	0.9%	2.44 [1.23, 3.66]
ZHAO Ruohan et al.2019	37.1	1.08	10	35.3	1.45	10	1.1%	1.35 [0.36, 2.34]
**Subtotal (95% CI)**			**476**			**476**	**21.9%**	**0.76 [0.30, 1.22]**
Heterogeneity: Tau² = 0.68; Chi² = 140.26, df = 14 (P < 0.00001); I² = 90%								
Test for overall effect: Z = 3.24 (P = 0.001)								
**2.1.4 Methionine analog**								
B.C.Benefield et al.2009	36.4	1	4	37.7	1	4	0.7%	-1.13 [-2.72, 0.46]
B.C.Benefield et al.2009	37.2	1	4	37.7	1	4	0.8%	-0.43 [-1.85, 0.98]
B.C.Benefield et al.2009	36.8	1	4	37.7	1	4	0.7%	-0.78 [-2.27, 0.70]
C.Wang et al.2010	28.5	2.52	15	26.5	2.52	15	1.4%	0.77 [0.03, 1.52]
F.X.Amaro et al.2022	39.7	4.65	15	38.4	4.65	15	1.4%	0.27 [-0.45, 0.99]
F.X.Amaro et al.2022	39.6	4.65	15	38.4	4.65	15	1.4%	0.25 [-0.47, 0.97]
F.X.Amaro et al.2022	39.5	4.65	15	38.4	4.65	15	1.4%	0.23 [-0.49, 0.95]
H.Rulquin et al.2006	31.5	2.8	4	31.4	2.8	4	0.8%	0.03 [-1.36, 1.42]
H.Rulquin et al.2006	31.8	2.8	4	31.4	2.8	4	0.8%	0.12 [-1.26, 1.51]
H.Rulquin et al.2006	32	2.8	4	31.4	2.8	4	0.8%	0.19 [-1.21, 1.58]
K.Xia et al.2012	29.54	2.56	10	26.57	2.56	10	1.2%	1.11 [0.15, 2.07]
K.Xia et al.2012	29.75	2.56	10	26.57	2.56	10	1.2%	1.19 [0.22, 2.16]
L.J.Erasmus et al.2013	35.03	3.13	10	35.02	3.13	10	1.3%	0.00 [-0.87, 0.88]
L.J.Erasmus et al.2013	35.17	3.13	10	35.02	3.13	10	1.3%	0.05 [-0.83, 0.92]
M.S.Piepenbrink et al.2004	45	4.4	16	42	4.4	16	1.4%	0.66 [-0.05, 1.38]
M.S.Piepenbrink et al.2004	41.9	4.4	16	42	4.4	16	1.5%	-0.02 [-0.72, 0.67]
N.R.St-Pierre et al.2005	40.7	4.76	15	39.8	4.76	15	1.4%	0.18 [-0.53, 0.90]
N.R.St-Pierre et al.2005	42.3	4.76	15	39.8	4.76	15	1.4%	0.51 [-0.22, 1.24]
N.R.St-Pierre et al.2005	43.2	4.76	15	39.8	4.76	15	1.4%	0.69 [-0.05, 1.44]
PEI Mingcai et al.2023	38.77	1.11	20	37.93	0.71	20	1.5%	0.88 [0.23, 1.54]
R.S.Ordway et al.2009	43.5	4.2	18	43.4	4.43	20	1.5%	0.02 [-0.61, 0.66]
R.S.Ordway et al.2009	42	4.32	19	43.4	4.43	20	1.5%	-0.31 [-0.95, 0.32]
S.E.Räisänen et al.2022	50.1	3.8	6	51.2	3.8	6	1.0%	-0.27 [-1.41, 0.87]
S.J.Whelan et al.2011	26.8	0.55	2	25.2	0.55	2	0.0%	1.66 [-7.95, 11.27]
S.L.Liang et al. 2019	31.91	2.22	12	31.51	2.22	12	1.3%	0.17 [-0.63, 0.98]
S.L.Liang et al. 2019	33.23	2.22	12	31.51	2.22	12	1.3%	0.75 [-0.08, 1.58]
S.L.Liang et al. 2019	31.43	2.22	12	31.51	2.22	12	1.3%	-0.03 [-0.84, 0.77]
YANG Baokui et al.2017	34.32	2.73	12	27.49	4.51	12	1.2%	1.77 [0.80, 2.74]
Z.H.Chen et al.2011	42.1	3.44	14	41.8	3.44	14	0.0%	0.08 [-0.66, 0.83]
**Subtotal (95% CI)**			**314**			**317**	**33.1%**	**0.33 [0.13, 0.52]**
Heterogeneity: Tau² = 0.07; Chi² = 37.15, df = 27 (P = 0.09); I² = 27%								
Test for overall effect: Z = 3.30 (P = 0.0010)								
**Total (95% CI)**			**1568**			**1566**	**100.0%**	**0.44 [0.28, 0.60]**
Heterogeneity: Tau² = 0.32; Chi² = 299.67, df = 78 (P < 0.00001); I² = 74%								
Test for overall effect: Z = 5.45 (P < 0.00001)								
Test for subgroup differences: Chi² = 3.05, df = 3 (P = 0.38), I² = 1.7%								

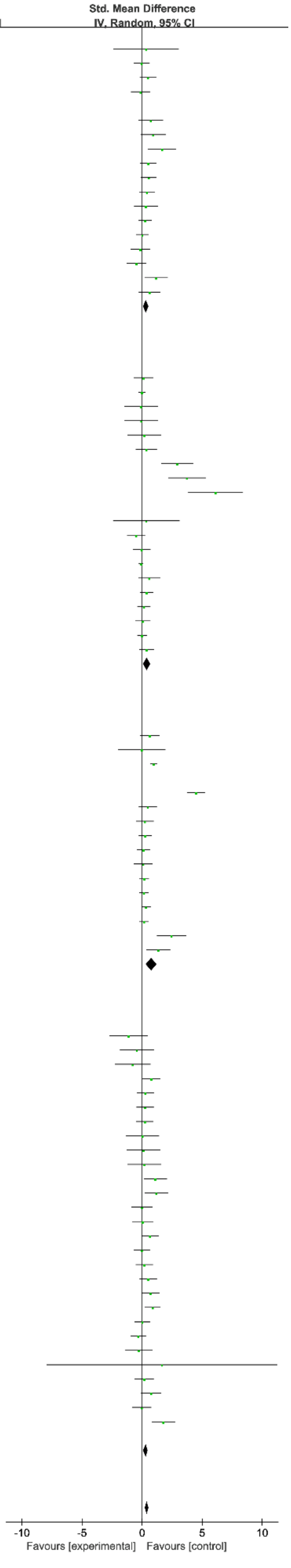

Supplementary Figure S6: Forest plot of milk yield for different amino acid categories in Holstein dairy cows.

Study or Subgroup	Experimental Mean	SD	Total	Control Mean	SD	Total	Weight	Std. Mean Difference IV, Random, 95% CI
**2.2.1 RPLys**								
C.Lee et al.2015	3.3	0.21	2	3.47	0.21	2	0.2%	-0.46 [-3.73, 2.81]
D.D.Girma et al.2019	4.31	1.07	17	3.91	1.07	17	1.5%	0.37 [-0.31, 1.04]
D.D.Girma et al.2019	3.65	1.07	17	4.29	1.07	17	1.5%	-0.58 [-1.27, 0.10]
F.Giallongo et al.2016	3.84	0.43	12	3.72	0.43	12	1.3%	0.27 [-0.54, 1.07]
J.Třináctý et al.2009	3.065	0.2522	1	3.59	0.2522	1		Not estimable
JIA Ming-jun et al.2010	4.431	0.37	8	4.392	0.28	8	1.1%	0.11 [-0.87, 1.09]
JIA Ming-jun et al.2010	4.4	0.42	8	4.392	0.28	8	1.1%	0.02 [-0.96, 1.00]
JIA Ming-jun et al.2010	4.551	0.38	8	4.392	0.28	8	1.1%	0.45 [-0.55, 1.45]
L.K.Fehlberg et al.2020	4.44	0.68	18	4.31	0.68	18	1.5%	0.19 [-0.47, 0.84]
L.K.Fehlberg et al.2020	4.56	0.7	19	4.31	0.68	18	1.5%	0.35 [-0.30, 1.00]
L.K.Fehlberg et al.2020	4.59	0.72	20	4.31	0.68	18	1.5%	0.39 [-0.25, 1.03]
Nelson E.Lobos et al.2021	4.11	0.27	8	4.19	0.27	8	1.1%	-0.28 [-1.27, 0.71]
P.Melendez et al.2023	4.1	0.56	26	4.14	0.56	26	1.7%	-0.07 [-0.61, 0.47]
Pedro Melendez et al.2023	4.49	0.39	26	4.63	0.39	26	1.7%	-0.35 [-0.90, 0.19]
V.M.R.Malacco et al.2021	3.28	0.38	12	3.47	0.38	12	1.3%	-0.48 [-1.30, 0.33]
V.M.R.Malacco et al.2021	3.4	0.38	12	3.47	0.38	12	1.3%	-0.18 [-0.98, 0.62]
Xiaoshi Wei et al.2023	4.09	0.36	10	3.96	0.36	10	1.2%	0.35 [-0.54, 1.23]
Xiaoshi Wei et al.2023	4.01	0.36	10	3.42	0.36	10	1.0%	1.57 [0.54, 2.60]
**Subtotal (95% CI)**			**234**			**231**	**21.3%**	**0.08 [-0.13, 0.30]**
Heterogeneity: Tau² = 0.04; Chi² = 20.59, df = 16 (P = 0.19); I² = 22%								
Test for overall effect: Z = 0.73 (P = 0.46)								
**2.2.2 RPMet**								
F.Giallongo et al.2016	3.8	0.43	12	3.72	0.43	12	1.3%	0.18 [-0.62, 0.98]
G.F.M.Leão et al.2024	4.45	1	83	4.1	1	83	2.0%	0.35 [0.04, 0.65]
HAN Zhao-yu et al.2007	3.99	0.5	4	3.72	0.4	4	0.7%	0.52 [-0.91, 1.95]
HAN Zhao-yu et al.2007	3.73	0.4	4	3.72	0.4	4	0.7%	0.02 [-1.36, 1.41]
HAN Zhao-yu et al.2007	3.86	0.4	4	3.72	0.4	4	0.7%	0.30 [-1.10, 1.71]
Hosam H.Titi et al.2013	3.49	0.05	10	3.42	0.05	10	1.1%	1.34 [0.35, 2.33]
Hosam H.Titi et al.2013	3.61	0.05	10	3.42	0.05	10	0.6%	3.64 [2.11, 5.17]
Hosam H.Titi et al.2013	3.54	0.05	10	3.48	0.05	10	1.1%	1.15 [0.19, 2.11]
Hosam H.Titi et al.2013	3.55	0.05	10	3.48	0.05	10	1.1%	1.34 [0.35, 2.33]
J.Třináctý et al.2009	3.191	0.2522	1	3.59	0.2522	1		Not estimable
K.Zhao et al.2019	4.16	0.3	2	3.95	0.3	2	0.2%	0.40 [-2.59, 3.39]
M.T.Socha et al.2005	3.88	0.22	14	3.62	0.22	14	1.3%	1.15 [0.34, 1.96]
M.T.Socha et al.2005	3.63	0.22	14	3.69	0.22	14	1.4%	-0.26 [-1.01, 0.48]
Mateus Z.Toledo et al.2021	3.87	0.44	217	3.77	0.44	213	2.1%	0.23 [0.04, 0.42]
MEHRNAZ ARDALAN et al.2010	3.3	0.09	10	3.29	0.09	10	1.2%	0.11 [-0.77, 0.98]
P.Melendez et al.2023	4.26	0.56	26	4.14	0.56	26	1.7%	0.21 [-0.33, 0.76]
Pedro Melendez et al.2023	4.52	0.39	26	4.63	0.39	26	1.7%	-0.28 [-0.82, 0.27]
Tetsuo Tamura et al.2019	3.74	0.19	19	3.61	0.18	20	1.5%	0.69 [0.04, 1.34]
Yuanxiao Li et al.2022	3.8	0.21	47	3.49	0.21	49	1.8%	1.46 [1.01, 1.92]
Z.Zhou et al.2016	3.75	0.69	21	3.61	0.67	20	1.6%	0.20 [-0.41, 0.82]
**Subtotal (95% CI)**			**544**			**542**	**23.6%**	**0.57 [0.28, 0.87]**
Heterogeneity: Tau² = 0.24; Chi² = 65.86, df = 18 (P < 0.00001); I² = 73%								
Test for overall effect: Z = 3.84 (P = 0.0001)								
**2.2.3 RPLys+RPMet**								
C.Lee et al.2012	3.32	0.41	12	3.51	0.41	12	1.3%	-0.45 [-1.26, 0.36]
C.Lee et al.2015	3.41	0.21	2	3.47	0.21	2	0.4%	-0.16 [-2.33, 2.00]
Emrah GÜLGÜN et.al.2022	3.87	0.2	100	3.77	0.2	100	2.0%	0.50 [0.22, 0.78]
J.Třináctý et al.2009	3.372	0.2522	1	3.59	0.2522	1		Not estimable
Lu Na et al.2019	4.26	0.13	48	4.26	0.1	48	1.9%	0.00 [-0.40, 0.40]
M.T.Socha et al.2005	3.74	0.22	14	3.69	0.22	14	1.4%	0.22 [-0.52, 0.96]
M.T.Socha et al.2005	3.65	0.22	14	3.62	0.22	14	1.4%	0.13 [-0.61, 0.87]
P.Melendez et al.2023	4.05	0.56	26	4.14	0.56	26	1.7%	-0.16 [-0.70, 0.39]
Pedro Melendez et al.2023	4.68	0.39	26	4.63	0.39	26	1.7%	0.13 [-0.42, 0.67]
T.Vanden Bossche et al.2023	4.35	0.29	13	4.34	0.29	13	1.3%	0.03 [-0.74, 0.80]
Xu Yuannian et al.2007	3.36	0.31	50	3.31	0.46	50	1.9%	0.13 [-0.27, 0.52]
Xu Yuannian et al.2007	3.48	0.77	50	3.31	0.46	50	1.9%	0.27 [-0.13, 0.66]
Xu Yuannian et al.2007	3.35	0.34	50	3.24	0.31	50	1.9%	0.34 [-0.06, 0.73]
Xu Yuannian et al.2007	3.13	0.26	50	3.57	0.47	50	1.8%	-1.15 [-1.57, -0.73]
ZHAO Ruohan et al.2019	3.44	0.15	10	3.89	0.12	10	0.7%	-3.17 [-4.58, -1.77]
ZHAO Ruohan et al.2019	3.59	0.16	10	3.89	0.12	10	0.9%	-2.03 [-3.16, -0.91]
**Subtotal (95% CI)**			**476**			**476**	**22.0%**	**-0.21 [-0.55, 0.14]**
Heterogeneity: Tau² = 0.33; Chi² = 79.40, df = 14 (P < 0.00001); I² = 82%								
Test for overall effect: Z = 1.18 (P = 0.24)								
**2.2.4 Methionine analog**								
B.C.Benefield et al.2009	3.3	0.08	4	3.21	0.08	4	0.6%	0.98 [-0.56, 2.52]
B.C.Benefield et al.2009	3.35	0.08	4	3.21	0.08	4	0.5%	1.52 [-0.21, 3.26]
B.C.Benefield et al.2009	3.33	0.08	4	3.21	0.08	4	0.6%	1.30 [-0.35, 2.96]
C.Wang et al.2010	3.95	0.19	15	3.6	0.19	15	1.2%	1.79 [0.93, 2.66]
F.X.Amaro et al.2022	3.54	0.54	15	3.54	0.54	15	1.4%	0.00 [-0.72, 0.72]
F.X.Amaro et al.2022	3.31	0.54	15	3.54	0.54	15	1.4%	-0.41 [-1.14, 0.31]
F.X.Amaro et al.2022	3.3	0.54	15	3.54	0.54	15	1.4%	-0.43 [-1.16, 0.29]
H.Rulquin et al.2006	4.2	0.3	4	4.16	0.3	4	0.7%	0.12 [-1.27, 1.50]
H.Rulquin et al.2006	4.26	0.3	4	4.16	0.3	4	0.7%	0.29 [-1.11, 1.69]
H.Rulquin et al.2006	4.16	0.3	4	4.16	0.3	4	0.7%	0.00 [-1.39, 1.39]
K.Xia et al.2012	3.78	0.149	10	3.69	0.149	10	1.2%	0.58 [-0.32, 1.48]
K.Xia et al.2012	3.89	0.149	10	3.69	0.149	10	1.1%	1.29 [0.30, 2.27]
L.J.Erasmus et al.2013	3.93	0.44	10	3.84	0.44	10	1.2%	0.20 [-0.68, 1.08]
L.J.Erasmus et al.2013	4.27	0.44	10	3.84	0.44	10	1.1%	0.94 [0.00, 1.87]
M.S.Piepenbrink et al.2004	4	0.36	16	4.2	0.36	16	1.4%	-0.54 [-1.25, 0.17]
M.S.Piepenbrink et al.2004	4.08	0.36	16	4.2	0.36	16	1.4%	-0.32 [-1.02, 0.37]
N.R.St-Pierre et al.2005	3.76	0.43	15	3.61	0.43	15	1.4%	0.34 [-0.38, 1.06]
N.R.St-Pierre et al.2005	3.82	0.43	15	3.61	0.43	15	1.4%	0.48 [-0.25, 1.20]
N.R.St-Pierre et al.2005	3.86	0.43	15	3.61	0.43	15	1.4%	0.57 [-0.17, 1.30]
PEI Mingcai et al.2023	3.88	0.05	20	3.8	0.05	20	1.4%	1.57 [0.85, 2.29]
R.S.Ordway et al.2009	3.65	0.31	18	3.65	0.33	20	1.5%	0.00 [-0.64, 0.64]
R.S.Ordway et al.2009	3.68	0.32	19	3.65	0.33	20	1.5%	0.09 [-0.54, 0.72]
S.E.Räisänen et al.2022	3.47	0.4	6	3.77	0.4	6	0.9%	-0.69 [-1.87, 0.49]
S.J.Whelan et al.2011	3.79	0.13	2	3.76	0.13	2	0.4%	0.13 [-1.97, 2.23]
S.L.Liang et al. 2019	3.98	0.45	12	4	0.45	12	1.3%	-0.04 [-0.84, 0.76]
S.L.Liang et al. 2019	4.05	0.45	12	4	0.45	12	1.3%	0.11 [-0.69, 0.91]
S.L.Liang et al. 2019	3.95	0.45	12	4	0.45	12	1.3%	-0.11 [-0.91, 0.69]
YANG Baokui et al.2017	3.84	0.08	12	3.73	0.04	12	1.1%	1.68 [0.73, 2.63]
Z.H.Chen et al.2011	3.93	0.41	14	3.52	0.41	14	1.3%	0.97 [0.18, 1.76]
**Subtotal (95% CI)**			**328**			**331**	**33.0%**	**0.38 [0.12, 0.64]**
Heterogeneity: Tau² = 0.28; Chi² = 68.04, df = 28 (P < 0.0001); I² = 59%								
Test for overall effect: Z = 2.86 (P = 0.004)								
**Total (95% CI)**			**1582**			**1580**	**100.0%**	**0.24 [0.10, 0.38]**
Heterogeneity: Tau² = 0.25; Chi² = 255.64, df = 79 (P < 0.00001); I² = 69%								
Test for overall effect: Z = 3.28 (P = 0.001)								
Test for subgroup differences: Chi² = 14.65, df = 3 (P = 0.002), I² = 79.5%								

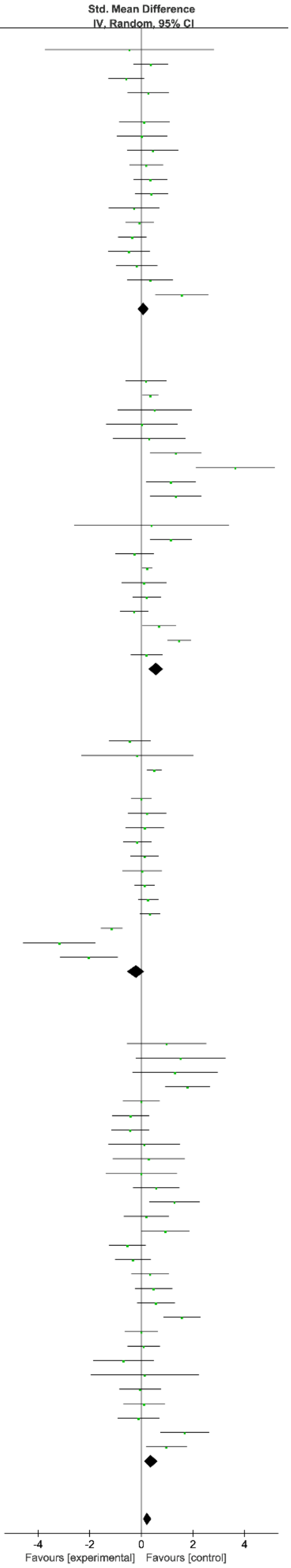

Supplementary Figure S7: Forest plot of milk fat for different amino acid categories in Holstein dairy cows.

Study or Subgroup	Experimental Mean	Experimental SD	Experimental Total	Control Mean	Control SD	Control Total	Weight	Std. Mean Difference IV, Random, 95% CI
**2.3.1 RPLys**								
C.Lee et al.2015	4.64	0.15	2	4.65	0.15	2	0.6%	-0.04 [-2.01, 1.93]
D.D.Girma et al.2019	4.95	0.29	17	4.99	0.29	17	2.2%	-0.13 [-0.81, 0.54]
D.D.Girma et al.2019	4.93	0.29	17	4.74	0.29	17	2.2%	0.64 [-0.05, 1.33]
F.Giallongo et al.2016	4.8	0.14	12	4.78	0.14	12	1.9%	0.14 [-0.66, 0.94]
J.Třináctý et al.2009	5.002	0.0289	1	5.028	0.0289	1		Not estimable
JIA Ming-jun et al.2010	4.758	0.05	8	4.716	0.06	8	1.5%	0.72 [-0.30, 1.74]
JIA Ming-jun et al.2010	4.768	0.07	8	4.716	0.06	8	1.5%	0.75 [-0.27, 1.78]
JIA Ming-jun et al.2010	4.777	0.07	8	4.716	0.06	8	1.4%	0.88 [-0.16, 1.93]
L.K.Fehlberg et al.2020	4.77	0.17	18	4.78	0.17	18	2.3%	-0.06 [-0.71, 0.60]
L.K.Fehlberg et al.2020	4.74	0.17	19	4.78	0.17	18	2.3%	-0.23 [-0.88, 0.42]
L.K.Fehlberg et al.2020	4.73	0.18	20	4.78	0.17	18	2.3%	-0.28 [-0.92, 0.36]
Nelson E.Lobos et al.2021	4.91	0.11	8	4.92	0.11	8	1.5%	-0.09 [-1.07, 0.89]
V.M.R.Malacco et al.2021	4.89	0.1	12	4.88	0.1	12	1.9%	0.10 [-0.70, 0.90]
V.M.R.Malacco et al.2021	4.81	0.1	12	4.88	0.1	12	1.8%	-0.68 [-1.50, 0.15]
Xiaoshi Wei et al.2023	5.15	0.07	10	5.13	0.07	10	1.7%	0.27 [-0.61, 1.16]
Xiaoshi Wei et al.2023	5.16	0.07	10	5.1	0.07	10	1.6%	0.82 [-0.10, 1.74]
**Subtotal (95% CI)**			**182**			**179**	**26.8%**	**0.12 [-0.11, 0.35]**
Heterogeneity: Tau² = 0.03; Chi² = 16.57, df = 14 (P = 0.28); I² = 16%								
Test for overall effect: Z = 1.04 (P = 0.30)								
**2.3.2 RPMet**								
F.Giallongo et al.2016	4.79	0.14	12	4.78	0.14	12	1.9%	0.07 [-0.73, 0.87]
G.F.M.Leão et al.2024	4.46	0.36	83	4.43	0.36	83	3.3%	0.08 [-0.22, 0.39]
J.Třináctý et al.2009	4.996	0.0289	1	5.028	0.0289	1		Not estimable
K.Zhao et al.2019	5.2	0.04	2	5.25	0.04	2	0.1%	-0.71 [-5.21, 3.78]
Mateus Z.Toledo et al.2021	4.86	0.15	217	4.88	0.15	213	3.5%	-0.13 [-0.32, 0.06]
MEHRNAZ ARDALAN et al.2010	4.81	0.16	10	4.77	0.16	10	1.7%	0.24 [-0.64, 1.12]
Tetsuo Tamura et al.2019	4.59	0.04	19	4.61	0.04	20	2.3%	-0.49 [-1.13, 0.15]
Yuanxiao Li et al.2022	4.87	0.07	47	4.85	0.07	49	3.0%	0.28 [-0.12, 0.69]
Z.Zhou et al.2016	4.69	0.18	21	4.71	0.18	20	2.4%	-0.11 [-0.72, 0.50]
**Subtotal (95% CI)**			**412**			**410**	**18.2%**	**-0.04 [-0.18, 0.10]**
Heterogeneity: Tau² = 0.00; Chi² = 6.54, df = 7 (P = 0.48); I² = 0%								
Test for overall effect: Z = 0.59 (P = 0.56)								
**2.3.3 RPLys+RPMet**								
C.Lee et al.2012	4.86	0.1	12	4.85	0.1	12	1.9%	0.10 [-0.70, 0.90]
C.Lee et al.2015	4.63	0.15	2	4.65	0.15	2	0.5%	-0.08 [-2.08, 1.93]
Emrah GÜLGÜN et.al.2022	4.88	0.3	100	4.85	0.3	100	3.3%	0.10 [-0.18, 0.38]
J.Třináctý et al.2009	4.991	0.0289	1	5.028	0.0289	1		Not estimable
T.Vanden Bossche et al.2023	4.69	0.07	13	4.69	0.07	13	2.0%	0.00 [-0.77, 0.77]
ZHAO Ruohan et al.2019	5.06	0.04	10	5.15	0.06	10	1.4%	-1.69 [-2.74, -0.64]
ZHAO Ruohan et al.2019	5.04	0.05	10	5.15	0.06	10	1.3%	-1.91 [-3.00, -0.81]
**Subtotal (95% CI)**			**148**			**148**	**10.5%**	**-0.52 [-1.21, 0.17]**
Heterogeneity: Tau² = 0.50; Chi² = 21.47, df = 5 (P = 0.0007); I² = 77%								
Test for overall effect: Z = 1.48 (P = 0.14)								
**2.3.4 Methionine analog**								
B.C.Benefield et al.2009	4.59	0.06	4	4.45	0.06	4	0.6%	2.03 [0.06, 4.00]
B.C.Benefield et al.2009	4.57	0.06	4	4.45	0.06	4	0.6%	1.74 [-0.09, 3.57]
B.C.Benefield et al.2009	4.54	0.06	4	4.45	0.06	4	0.7%	1.30 [-0.35, 2.96]
C.Wang et al.2010	5.04	0.16	15	5.01	0.16	15	2.1%	0.18 [-0.53, 0.90]
F.X.Amaro et al.2022	4.71	0.15	15	4.76	0.15	15	2.1%	-0.32 [-1.05, 0.40]
F.X.Amaro et al.2022	4.75	0.15	15	4.76	0.15	15	2.1%	-0.06 [-0.78, 0.65]
F.X.Amaro et al.2022	4.69	0.15	15	4.76	0.15	15	2.1%	-0.45 [-1.18, 0.27]
K.Xia et al.2012	4.91	0.06	10	4.94	0.06	10	1.7%	-0.48 [-1.37, 0.41]
K.Xia et al.2012	4.92	0.06	10	4.94	0.06	10	1.7%	-0.32 [-1.20, 0.56]
L.J.Erasmus et al.2013	4.43	0.19	10	4.65	0.19	10	1.6%	-1.11 [-2.07, -0.15]
L.J.Erasmus et al.2013	4.48	0.19	10	4.65	0.19	10	1.6%	-0.86 [-1.78, 0.07]
M.S.Piepenbrink et al.2004	4.69	0.16	16	4.7	0.16	16	2.2%	-0.06 [-0.75, 0.63]
M.S.Piepenbrink et al.2004	4.75	0.16	16	4.7	0.16	16	2.2%	0.30 [-0.39, 1.00]
N.R.St-Pierre et al.2005	4.83	0.12	15	4.79	0.12	15	2.1%	0.32 [-0.40, 1.05]
N.R.St-Pierre et al.2005	4.86	0.12	15	4.79	0.12	15	2.1%	0.57 [-0.16, 1.30]
N.R.St-Pierre et al.2005	4.91	0.12	15	4.79	0.12	15	2.0%	0.97 [0.21, 1.74]
PEI Mingcai et al.2023	4.96	0.03	20	4.85	0.04	20	1.6%	3.05 [2.11, 3.99]
R.S.Ordway et al.2009	4.84	0.08	18	4.84	0.08	20	2.3%	0.00 [-0.64, 0.64]
R.S.Ordway et al.2009	4.83	0.08	19	4.84	0.08	20	2.3%	-0.12 [-0.75, 0.51]
S.E.Räisänen et al.2022	4.86	0.11	6	4.87	0.11	6	1.3%	-0.08 [-1.22, 1.05]
S.J.Whelan et al.2011	4.7	0.03	2	4.68	0.03	2	0.3%	0.38 [-2.53, 3.29]
S.L.Liang et al. 2019	5	0.1	12	5	0.1	12	1.9%	0.00 [-0.80, 0.80]
S.L.Liang et al. 2019	4.99	0.1	12	5	0.1	12	1.9%	-0.10 [-0.90, 0.70]
S.L.Liang et al. 2019	4.99	0.1	12	5	0.1	12	1.9%	-0.10 [-0.90, 0.70]
YANG Baokui et al.2017	4.84	0.07	12	4.65	0.09	12	1.4%	2.28 [1.21, 3.34]
Z.H.Chen et al.2011	4.83	0.19	14	4.79	0.19	14	2.0%	0.20 [-0.54, 0.95]
**Subtotal (95% CI)**			**316**			**319**	**44.5%**	**0.25 [-0.07, 0.57]**
Heterogeneity: Tau² = 0.46; Chi² = 87.62, df = 25 (P < 0.00001); I² = 71%								
Test for overall effect: Z = 1.54 (P = 0.12)								
**Total (95% CI)**			**1058**			**1056**	**100.0%**	**0.09 [-0.07, 0.25]**
Heterogeneity: Tau² = 0.18; Chi² = 137.35, df = 54 (P < 0.00001); I² = 61%								
Test for overall effect: Z = 1.08 (P = 0.28)								
Test for subgroup differences: Chi² = 5.88, df = 3 (P = 0.12), I² = 49.0%								

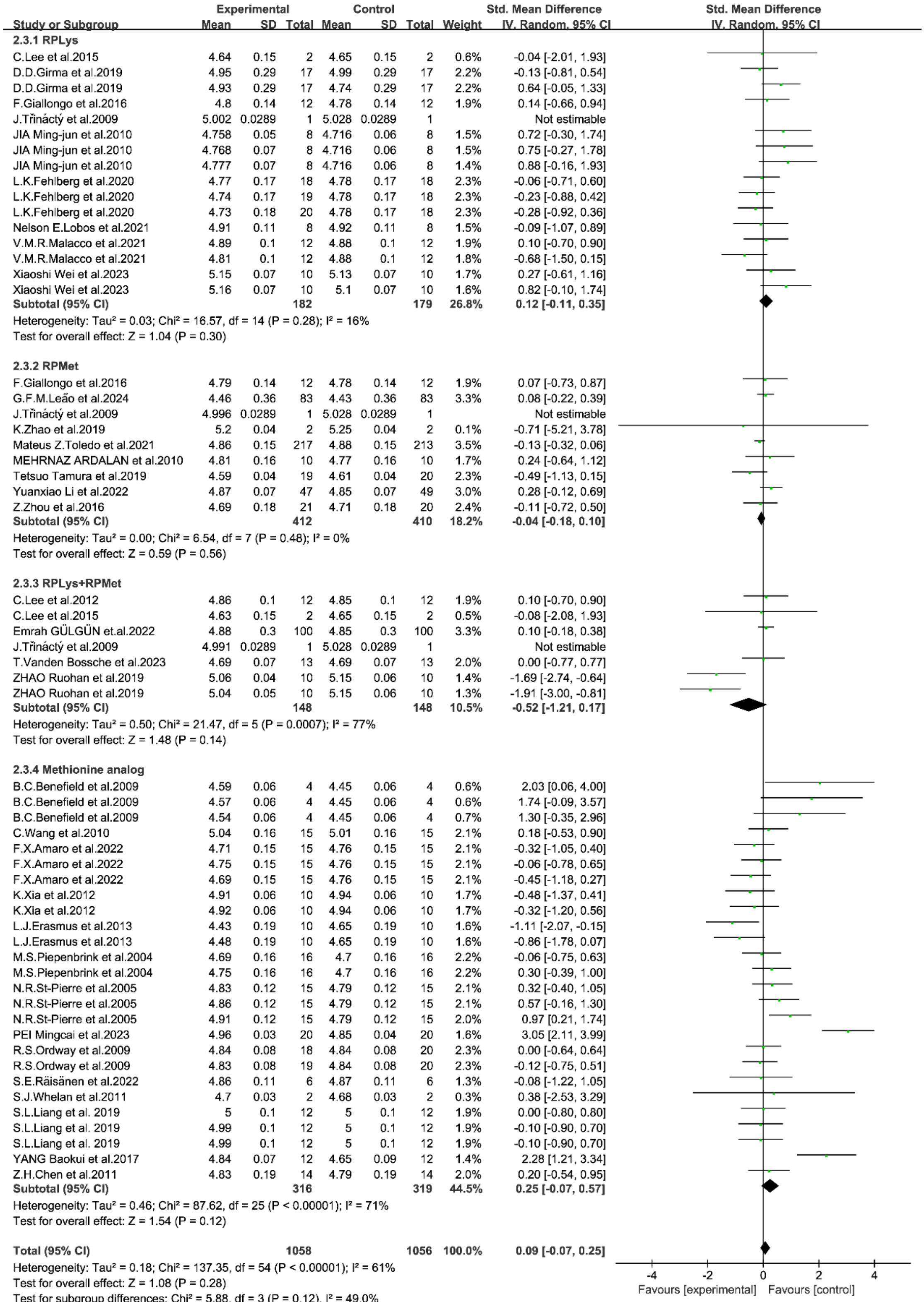

Supplementary Figure S8: Forest plot of milk lactose for different amino acid categories in Forest plot of milk fat for different amino acid categories in Holstein dairy cows.

Study or Subgroup	Experimental Mean	Experimental SD	Experimental Total	Control Mean	Control SD	Control Total	Weight	Std. Mean Difference IV, Random, 95% CI
**2.4.1 RPLys**								
D.D.Girma et al.2019	3.701	0.37	17	3.861	0.37	17	2.1%	-0.42 [-1.10, 0.26]
D.D.Girma et al.2019	3.611	0.37	17	3.71	0.37	17	2.1%	-0.26 [-0.94, 0.41]
J.Třináctý et al.2009	3.081	0.0478	1	2.971	0.0478	1		Not estimable
JIA Ming-jun et al.2010	3.09	0.04	8	3.061	0.05	8	1.6%	0.61 [-0.40, 1.61]
JIA Ming-jun et al.2010	3.097	0.04	8	3.061	0.05	8	1.6%	0.75 [-0.27, 1.78]
JIA Ming-jun et al.2010	3.127	0.04	8	3.061	0.05	8	1.5%	1.38 [0.25, 2.50]
L.K.Fehlberg et al.2020	3.31	0.21	18	3.32	0.21	18	2.2%	-0.05 [-0.70, 0.61]
L.K.Fehlberg et al.2020	3.24	0.22	19	3.32	0.21	18	2.2%	-0.36 [-1.01, 0.29]
L.K.Fehlberg et al.2020	3.32	0.22	20	3.32	0.21	18	2.2%	0.00 [-0.64, 0.64]
P.Melendez et al.2023	3.23	0.2	26	3.27	0.2	26	2.3%	-0.20 [-0.74, 0.35]
Pedro Melendez et al.2023	3.56	0.3	26	3.45	0.3	26	2.3%	0.36 [-0.19, 0.91]
V.M.R.Malacco et al.2021	3.02	0.12	12	2.99	0.12	12	1.9%	0.24 [-0.56, 1.05]
V.M.R.Malacco et al.2021	3.06	0.12	12	2.99	0.12	12	1.9%	0.56 [-0.26, 1.38]
Xiaoshi Wei et al.2023	3.29	0.15	10	3.13	0.15	10	1.7%	1.02 [0.08, 1.97]
Xiaoshi Wei et al.2023	3.38	0.15	10	3.25	0.15	10	1.8%	0.83 [-0.09, 1.75]
**Subtotal (95% CI)**			**212**			**209**	**27.5%**	**0.20 [-0.06, 0.47]**
Heterogeneity: Tau² = 0.10; Chi² = 22.30, df = 13 (P = 0.05); I² = 42%								
Test for overall effect: Z = 1.52 (P = 0.13)								
**2.4.2 RPMet**								
G.F.M.Leão et al.2024	3.47	0.36	83	3.39	0.36	83	2.6%	0.22 [-0.08, 0.53]
HAN Zhao-yu et al.2007	3.62	0.3	4	3.53	0.3	4	1.2%	0.26 [-1.14, 1.66]
HAN Zhao-yu et al.2007	3.63	0.4	4	3.53	0.3	4	1.2%	0.25 [-1.15, 1.64]
HAN Zhao-yu et al.2007	3.59	0.2	4	3.53	0.3	4	1.2%	0.20 [-1.19, 1.60]
Hosam H.Titi et al.2013	3.1	0.07	10	3.1	0.07	10	1.8%	0.00 [-0.88, 0.88]
Hosam H.Titi et al.2013	3.03	0.07	10	3.04	0.07	10	1.8%	-0.14 [-1.01, 0.74]
Hosam H.Titi et al.2013	3.08	0.07	10	3.04	0.07	10	1.8%	0.55 [-0.35, 1.44]
Hosam H.Titi et al.2013	3.13	0.07	10	3.1	0.07	10	1.8%	0.41 [-0.48, 1.30]
J.Třináctý et al.2009	3.012	0.0478	1	2.971	0.0478	1		Not estimable
K.Zhao et al.2019	3.65	0.13	2	3.48	0.13	2	0.2%	0.75 [-3.91, 5.41]
Mateus Z.Toledo et al.2021	3.07	0.15	217	2.95	0.15	213	2.7%	0.80 [0.60, 1.00]
MEHRNAZ ARDALAN et al.2010	3.16	0.06	10	3.1	0.06	10	1.7%	0.96 [0.02, 1.89]
P.Melendez et al.2023	3.28	0.2	26	3.27	0.2	26	2.3%	0.05 [-0.49, 0.59]
Pedro Melendez et al.2023	3.47	0.3	26	3.45	0.3	26	2.3%	0.07 [-0.48, 0.61]
Tetsuo Tamura et al.2019	3.09	0.05	19	3.18	0.04	20	2.0%	-1.95 [-2.73, -1.18]
Yuanxiao Li et al.2022	3.34	0.03	47	3.39	0.03	49	2.4%	-1.65 [-2.12, -1.19]
Z.Zhou et al.2016	3.31	0.27	21	3.07	0.27	20	2.2%	0.87 [0.23, 1.52]
**Subtotal (95% CI)**			**504**			**502**	**29.3%**	**0.05 [-0.42, 0.51]**
Heterogeneity: Tau² = 0.67; Chi² = 133.04, df = 15 (P < 0.00001); I² = 89%								
Test for overall effect: Z = 0.20 (P = 0.84)								
**2.4.3 RPLys+RPMet**								
J.Třináctý et al.2009	3.078	0.0478	1	2.971	0.0478	1		Not estimable
Lu Na et al.2019	3.09	0.02	48	3.07	0.04	48	0.0%	0.63 [0.22, 1.04]
P.Melendez et al.2023	3.23	0.2	26	3.27	0.2	26	2.3%	-0.20 [-0.74, 0.35]
Pedro Melendez et al.2023	3.53	0.3	26	3.45	0.3	26	2.3%	0.26 [-0.28, 0.81]
T.Vanden Bossche et al.2023	3.74	0.11	13	3.75	0.11	13	2.0%	-0.09 [-0.86, 0.68]
Xu Yuannian et al.2007	2.73	0.28	50	2.84	0.26	50	2.5%	-0.40 [-0.80, -0.01]
Xu Yuannian et al.2007	2.78	0.22	50	2.91	0.28	50	2.5%	-0.51 [-0.91, -0.11]
Xu Yuannian et al.2007	2.72	0.23	50	2.84	0.27	50	2.5%	-0.47 [-0.87, -0.08]
Xu Yuannian et al.2007	2.84	0.22	50	2.94	0.27	50	2.5%	-0.40 [-0.80, -0.01]
ZHAO Ruohan et al.2019	3.14	0.06	10	3.13	0.07	10	1.8%	0.15 [-0.73, 1.02]
ZHAO Ruohan et al.2019	3.05	0.04	10	3.13	0.07	10	1.7%	-1.34 [-2.34, -0.35]
**Subtotal (95% CI)**			**286**			**286**	**20.2%**	**-0.33 [-0.54, -0.12]**
Heterogeneity: Tau² = 0.03; Chi² = 11.82, df = 8 (P = 0.16); I² = 32%								
Test for overall effect: Z = 3.06 (P = 0.002)								
**2.4.4 Methionine analog**								
C.Wang et al.2010	3.3	0.39	15	3.27	0.39	15	2.1%	0.07 [-0.64, 0.79]
K.Xia et al.2012	3.56	2.802	10	3.58	2.802	10	1.8%	-0.01 [-0.88, 0.87]
K.Xia et al.2012	3.51	2.802	10	3.58	2.802	10	1.8%	-0.02 [-0.90, 0.85]
L.J.Erasmus et al.2013	3.25	0.54	10	3.06	0.54	10	1.8%	0.34 [-0.55, 1.22]
L.J.Erasmus et al.2013	3.46	0.54	10	3.06	0.54	10	1.8%	0.71 [-0.20, 1.62]
PEI Mingcai et al.2023	3.14	0.03	20	3.11	0.03	20	2.1%	0.98 [0.32, 1.64]
R.S.Ordway et al.2009	2.81	0.13	18	2.72	0.14	20	2.2%	0.65 [-0.00, 1.31]
R.S.Ordway et al.2009	2.87	0.14	19	2.72	0.14	20	2.1%	1.05 [0.38, 1.72]
S.J.Whelan et al.2011	3.38	0.04	2	3.44	0.04	2	0.1%	-0.86 [-6.09, 4.37]
S.L.Liang et al. 2019	3.46	0.1	12	3.51	0.1	12	1.9%	-0.48 [-1.30, 0.33]
S.L.Liang et al. 2019	3.49	0.1	12	3.51	0.1	12	1.9%	-0.19 [-1.00, 0.61]
S.L.Liang et al. 2019	3.48	0.1	12	3.51	0.1	12	1.9%	-0.29 [-1.09, 0.52]
YANG Baokui et al.2017	3.19	0.04	12	3.05	0.05	12	1.4%	2.99 [1.76, 4.21]
**Subtotal (95% CI)**			**162**			**165**	**23.1%**	**0.43 [0.01, 0.84]**
Heterogeneity: Tau² = 0.37; Chi² = 36.91, df = 12 (P = 0.0002); I² = 67%								
Test for overall effect: Z = 2.01 (P = 0.04)								
**Total (95% CI)**			**1164**			**1162**	**100.0%**	**0.12 [-0.09, 0.32]**
Heterogeneity: Tau² = 0.38; Chi² = 247.05, df = 51 (P < 0.00001); I² = 79%								
Test for overall effect: Z = 1.13 (P = 0.26)								
Test for subgroup differences: Chi² = 15.48, df = 3 (P = 0.001), I² = 80.6%								

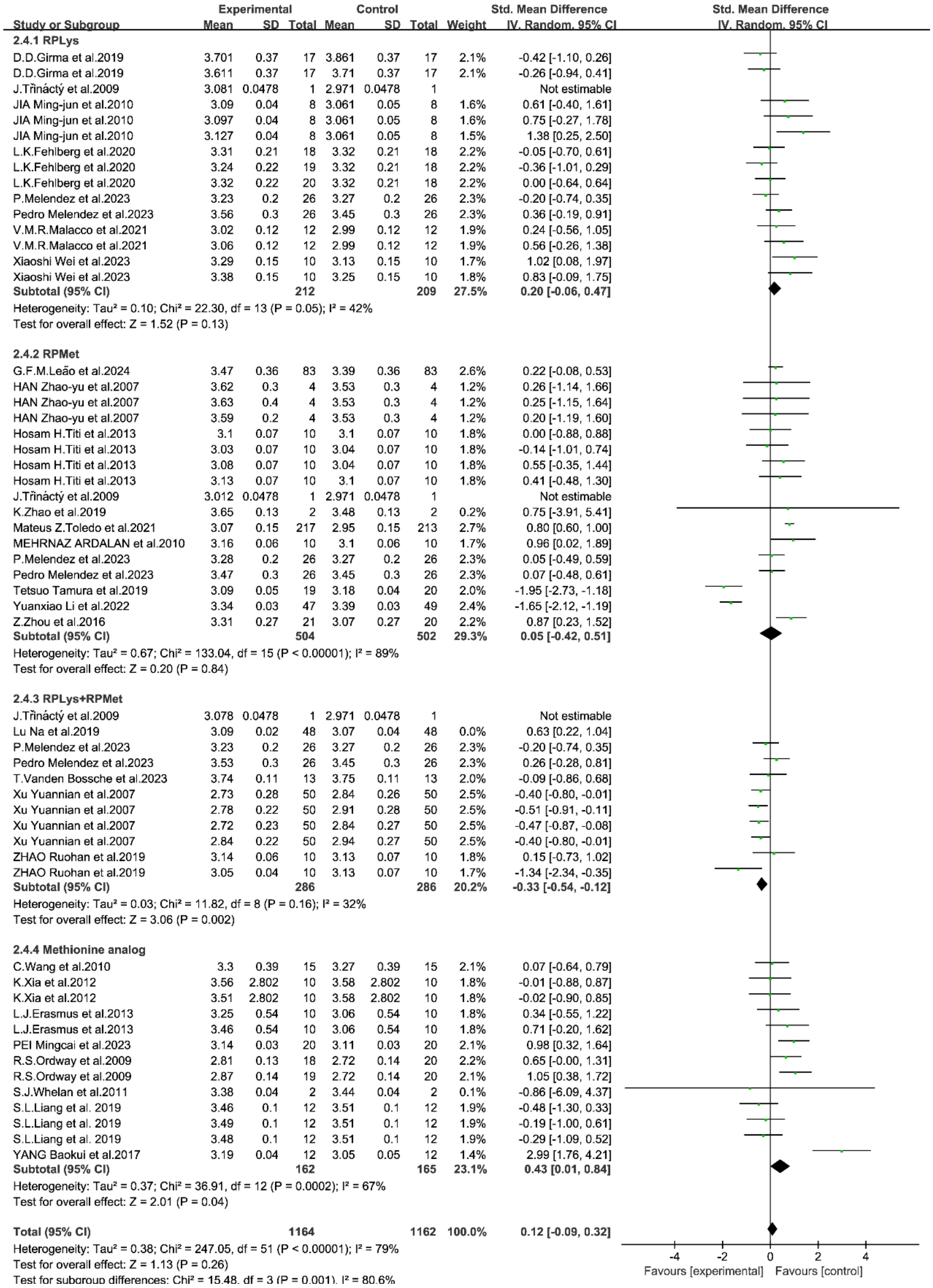

Supplementary Figure S9: Forest plot of milk protein for different amino acid categories in Holstein dairy cows.

Study or Subgroup	Experimental Mean	Experimental SD	Experimental Total	Control Mean	Control SD	Control Total	Weight	Std. Mean Difference IV, Random, 95% CI
**2.5.1 RPLys**								
C.Lee et al.2015	1.58	0.09	2	1.56	0.09	2	1.2%	0.13 [-1.96, 2.21]
C.Lee et al.2015	1.55	0.09	2	1.56	0.09	2	1.3%	-0.06 [-2.06, 1.93]
F.Giallongo et al.2016	1.29	0.1	12	1.27	0.1	12	2.6%	0.19 [-0.61, 1.00]
J.Třináctý et al.2009	1.28	0.065	1	1.48	0.065	1		Not estimable
J.Třináctý et al.2009	1.33	0.065	1	1.48	0.065	1		Not estimable
J.Třináctý et al.2009	1.41	0.065	1	1.48	0.065	1		Not estimable
L.K.Fehlberg et al.2020	2.44	0.61	19	2.67	0.59	18	2.8%	-0.37 [-1.03, 0.28]
L.K.Fehlberg et al.2020	2.51	0.63	20	2.67	0.59	18	2.8%	-0.26 [-0.90, 0.38]
L.K.Fehlberg et al.2020	2.37	0.59	18	2.67	0.59	18	2.8%	-0.50 [-1.16, 0.17]
Nelson E.Lobos et al.2021	1.69	0.11	8	1.65	0.11	8	2.3%	0.34 [-0.65, 1.33]
V.M.R.Malacco et al.2021	1.24	0.16	12	1.33	0.16	12	2.6%	-0.54 [-1.36, 0.27]
V.M.R.Malacco et al.2021	1.28	0.16	12	1.33	0.16	12	2.6%	-0.30 [-1.11, 0.50]
Xiaoshi Wei et al.2023	1.62	0.06	10	1.59	0.06	10	2.5%	0.48 [-0.41, 1.37]
Xiaoshi Wei et al.2023	1.61	0.06	10	1.63	0.06	10	2.5%	-0.32 [-1.20, 0.56]
**Subtotal (95% CI)**			**128**			**125**	**25.8%**	**-0.19 [-0.45, 0.06]**
Heterogeneity: Tau² = 0.00; Chi² = 6.30, df = 10 (P = 0.79); I² = 0%								
Test for overall effect: Z = 1.51 (P = 0.13)								
**2.5.2 RPMet**								
F.Giallongo et al.2016	1.28	0.1	12	1.27	0.1	12	2.6%	0.10 [-0.70, 0.90]
Hosam H.Titi et al.2013	1.01	0.03	10	1.02	0.03	10	2.5%	-0.32 [-1.20, 0.56]
Hosam H.Titi et al.2013	0.99	0.03	10	1.02	0.03	10	2.4%	-0.96 [-1.89, -0.02]
Hosam H.Titi et al.2013	0.91	0.03	10	0.99	0.03	10	2.0%	-2.55 [-3.80, -1.31]
Hosam H.Titi et al.2013	0.9	0.03	10	0.99	0.03	10	1.9%	-2.87 [-4.20, -1.55]
J.Třináctý et al.2009	1.33	0.065	1	1.48	0.065	1		Not estimable
J.Třináctý et al.2009	1.28	0.065	1	1.48	0.065	1		Not estimable
J.Třináctý et al.2009	1.41	0.065	1	1.48	0.065	1		Not estimable
K.Zhao et al.2019	1.31	0.1	2	1.23	0.1	2	0.6%	0.46 [-2.79, 3.70]
M.T.Socha et al.2005	1.94	0.11	14	1.85	0.11	14	2.6%	0.79 [0.02, 1.57]
M.T.Socha et al.2005	1.92	0.11	14	1.89	0.11	14	2.7%	0.26 [-0.48, 1.01]
Z.Zhou et al.2016	2.5	0.55	21	2.47	0.54	20	2.8%	0.05 [-0.56, 0.67]
**Subtotal (95% CI)**			**106**			**105**	**20.1%**	**-0.53 [-1.24, 0.18]**
Heterogeneity: Tau² = 0.88; Chi² = 41.10, df = 8 (P < 0.00001); I² = 81%								
Test for overall effect: Z = 1.45 (P = 0.15)								
**2.5.3 RPLys+RPMet**								
C.Lee et al.2012	1.41	0.11	12	1.4	0.11	12	2.6%	0.09 [-0.71, 0.89]
C.Lee et al.2015	1.52	0.08	2	1.55	0.08	2	1.0%	-0.21 [-2.52, 2.09]
Emrah GÜLGÜN et.al.2022	1.42	1.5	100	1.34	1.5	100	3.1%	0.05 [-0.22, 0.33]
J.Třináctý et al.2009	1.28	0.065	1	1.48	0.065	1		Not estimable
J.Třináctý et al.2009	1.33	0.065	1	1.48	0.065	1		Not estimable
J.Třináctý et al.2009	1.41	0.065	1	1.48	0.065	1		Not estimable
Lu Na et al.2019	1.86	0.01	48	1.78	0.02	48	2.6%	5.02 [4.19, 5.85]
M.T.Socha et al.2005	1.91	0.11	14	1.89	0.11	14	2.7%	0.18 [-0.57, 0.92]
M.T.Socha et al.2005	1.97	0.11	14	1.85	0.11	14	2.6%	1.06 [0.26, 1.86]
T.Vanden Bossche et al.2023	1.42	0.11	13	1.41	0.11	13	2.6%	0.09 [-0.68, 0.86]
**Subtotal (95% CI)**			**206**			**206**	**17.2%**	**0.94 [-0.30, 2.17]**
Heterogeneity: Tau² = 2.53; Chi² = 129.47, df = 6 (P < 0.00001); I² = 95%								
Test for overall effect: Z = 1.48 (P = 0.14)								
**2.5.4 Methionine analog**								
C.Wang et al.2010	1.38	0.12	15	1.27	0.12	15	2.6%	0.89 [0.14, 1.65]
F.X.Amaro et al.2022	1.66	0.23	15	1.55	0.23	15	2.7%	0.47 [-0.26, 1.19]
F.X.Amaro et al.2022	1.64	0.23	15	1.55	0.23	15	2.7%	0.38 [-0.34, 1.10]
F.X.Amaro et al.2022	1.75	0.23	15	1.55	0.23	15	2.7%	0.85 [0.09, 1.60]
N.R.St-Pierre et al.2005	1.78	0.27	15	1.79	0.27	15	2.7%	-0.04 [-0.75, 0.68]
N.R.St-Pierre et al.2005	1.78	0.27	15	1.79	0.27	15	2.7%	-0.04 [-0.75, 0.68]
N.R.St-Pierre et al.2005	1.89	0.27	15	1.79	0.27	15	2.7%	0.36 [-0.36, 1.08]
R.S.Ordway et al.2009	1.92	0.14	18	2	0.14	20	2.8%	-0.56 [-1.21, 0.09]
R.S.Ordway et al.2009	1.98	0.14	19	2	0.14	20	2.8%	-0.14 [-0.77, 0.49]
S.E.Räisänen et al.2022	2.08	0.11	6	2.05	0.11	6	2.1%	0.25 [-0.89, 1.39]
S.L.Liang et al. 2019	1.33	0.1	12	1.36	0.1	12	2.6%	-0.29 [-1.09, 0.52]
S.L.Liang et al. 2019	1.4	0.1	12	1.36	0.1	12	2.6%	0.39 [-0.42, 1.20]
S.L.Liang et al. 2019	1.31	0.1	12	1.36	0.1	12	2.6%	-0.48 [-1.30, 0.33]
Z.H.Chen et al.2011	1.59	0.15	14	1.54	0.15	14	2.7%	0.32 [-0.42, 1.07]
**Subtotal (95% CI)**			**198**			**201**	**36.9%**	**0.16 [-0.08, 0.39]**
Heterogeneity: Tau² = 0.06; Chi² = 18.38, df = 13 (P = 0.14); I² = 29%								
Test for overall effect: Z = 1.28 (P = 0.20)								
**Total (95% CI)**			**638**			**637**	**100.0%**	**0.09 [-0.20, 0.37]**
Heterogeneity: Tau² = 0.66; Chi² = 217.35, df = 40 (P < 0.00001); I² = 82%								
Test for overall effect: Z = 0.60 (P = 0.55)								
Test for subgroup differences: Chi² = 8.17, df = 3 (P = 0.04), I² = 63.3%								

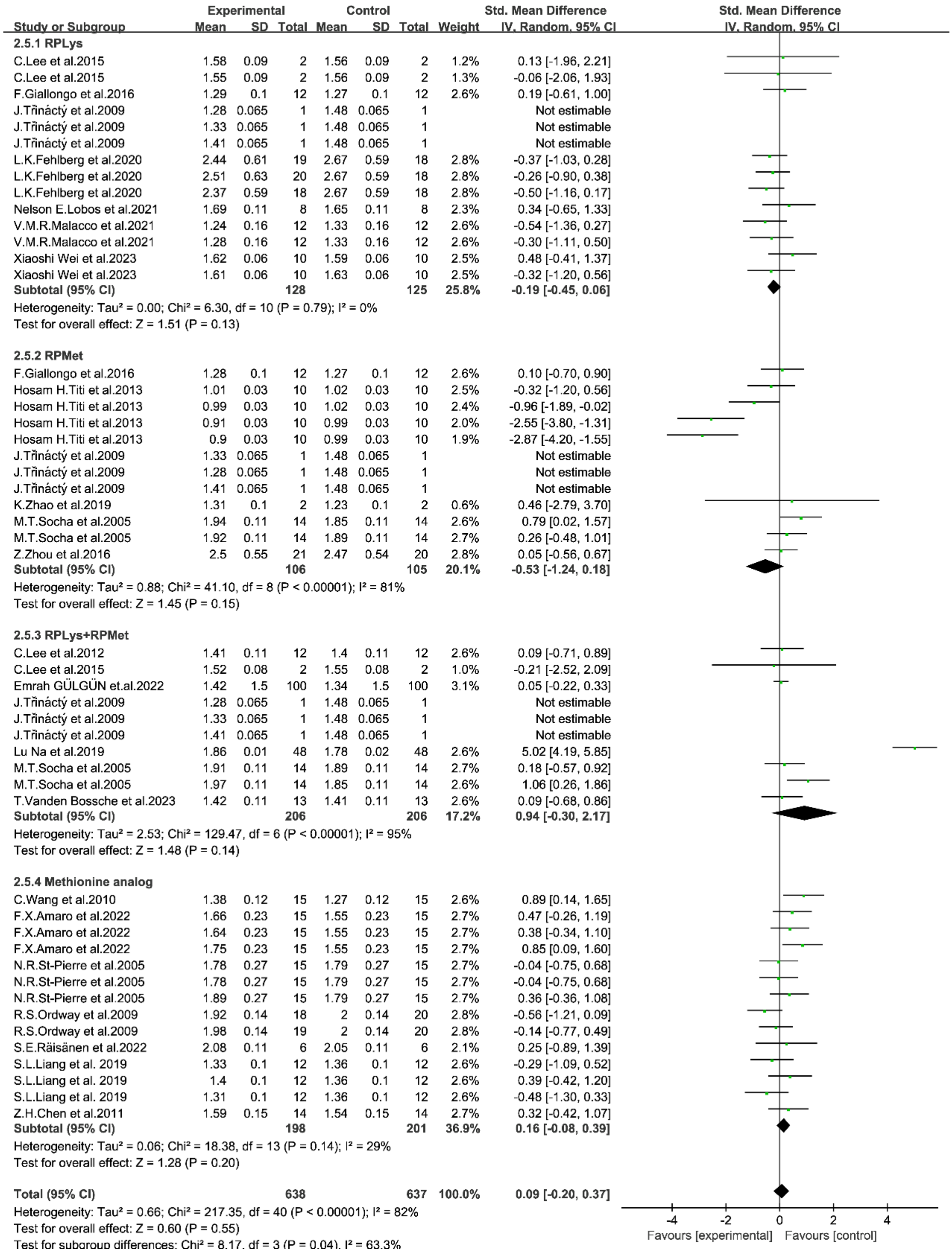

Supplementary Figure S10: Forest plot of Feed conversion-related traits for different amino acid categories in Holstein dairy cows.
